# Acoustic cues into a surgeon-assist physical AI for detecting bone penetration during spinal surgery

**DOI:** 10.1038/s41598-026-48857-w

**Published:** 2026-04-19

**Authors:** Hideaki Fujiwara, Takahito Fujimori, Yuya Kanie, Masayuki Furuya, Hirotatsu Imai, Koki Hosozawa, Kosuke Kita, Koki Kishimoto, Kei Shinyashiki, Yuichiro Ukon, Seiji Okada

**Affiliations:** 1 Department of Orthopaedic Surgery, Hoshigaoka Medical Center, Osaka, Japan; 2 Department of Orthopaedic Surgery, Graduate School of Medicine, The University of Osaka, Yamadaoka 2–2, Suita city, 565–0871 Osaka Japan; 3 Strategic Global Partnership and X-Innovation Initiative, Graduate School of Medicine, The University of Osaka, Osaka, Japan

**Keywords:** Acoustic analysis, Bone penetration, Spinal decompression, Machine learning, Physical AI, Surgeon-assist, Intraoperative sound, Diagnostic accuracy, Gradient boosting, Precision–recall analysis, Surgical skill quantification, Engineering, Health care, Medical research

## Abstract

Skilled surgeons can detect subtle changes in bone-cutting sounds to recognize bone penetration, but acoustic cues are subjective and dependent on surgical experience. The objective of this study was to develop an artificial intelligence (AI) model capable of detecting bone penetration from intraoperative percussion sounds. A total of 1,236 chisel strikes were identified from intraoperative recordings obtained during lumbar and thoracic spinal decompression surgeries performed using a chisel, and were labeled as penetration or non-penetration. Acoustic features were extracted per strike and expanded across 3-hit sliding windows to capture dynamic temporal changes. A gradient boosting machine learning classifier (LightGBM) was trained, and 10% of the data were reserved as an independent test set. Model performance was primarily evaluated using the area under the precision–recall curve (PR-AUC) and receiver operating characteristic AUC (ROC-AUC). On the independent test set, the model yielded a ROC-AUC of 0.838 and a PR-AUC of 0.604. In a sensitivity analysis, the model achieved a sensitivity of 0.828, specificity of 0.767, accuracy 0.784, F1-score 0.686, and precision 0.585. Feature importance analysis revealed that dynamic changes between consecutive strikes, such as mel-frequency cepstral coefficients (MFCCs), zero-crossing rate, and spectral contrast, were the most influential predictors. An AI model analyzing intraoperative percussion sounds demonstrated a robust ability to detect bone penetration, with consistent ROC performance and clinically acceptable PR-AUC. Quantifying acoustic cues traditionally interpreted subjectively by surgeons may support intraoperative decision-making and surgical training.

**Supplementary Information:**

The online version contains supplementary material available at 10.1038/s41598-026-48857-w.

## Introduction

Sound is an important source of information, especially when visual inspection is not possible. Auscultation is a representative example, where physicians assess internal organ conditions by listening to breath and heart sounds. In orthopedics as well, it is known that before the invention of X-rays, fractures were diagnosed by percussion sounds^1,2^.

Laminectomy using a chisel is one of the fundamental procedures in spinal decompression surgery^3^. During percussion with a chisel, the surgeon integrates multiple sensory cues—such as resistance of the chisel, speed, and progression—to determine whether the bone has been cut^4^. One of the key pieces of information for this judgment is the change in the percussion sound. This principle is similar to that observed in arthroplasty, where the sound changes as the prosthesis becomes firmly seated in the joint^1,5,6^. Typically, perceiving these sound changes requires surgical experience; however, recent studies have begun to explore the possibility of using artificial intelligence (AI) to replicate human ability and other bone-related diagnostic or predictive applications^7–12^.

In industry, acoustic AI has been applied to detecting delamination or cracks in concrete and tiles and to identifying machine tool faults^13^. In medicine, similar approaches have been used to analyze respiratory and heart sounds for abnormality detection^14,15^. In this study, we use the term Physical AI to describe AI systems that interpret real-world physical signals—such as sound, vibration, force, or motion—captured by sensors to support or automate task execution. This is an emerging research field^16,17^.

We aimed to extend this concept to the surgical domain. We use the term Surgeon-assist Physical AI (SPAI) to refer specifically to Physical AI systems designed to augment surgeons’ intraoperative sensory judgment and decision-making. The objective of this study was to construct an AI model that can determine whether bone has been cut based on percussion sounds during laminectomy and to evaluate its performance.

## Methods

**Fig. 1:**
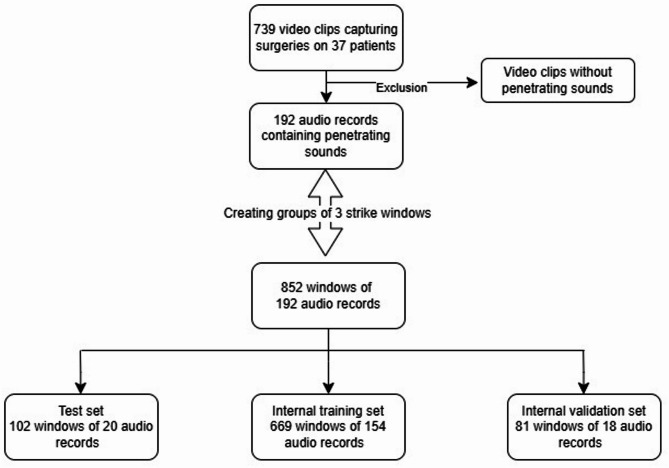
Flow diagram of data selection and partitioning. A total of 739 video clips from 37 patients were collected. Clips without penetrating sounds were excluded, leaving 192 audio records. From these, 852 three-strike windows were generated. The dataset was divided into an independent test set (102 windows from 20 audio records) and a training dataset (750 windows from 172 audio records). The training dataset was further split into an internal training set (669 windows from 154 audio records) and an internal validation set (81 windows from 18 audio records).

**Fig. 2:**
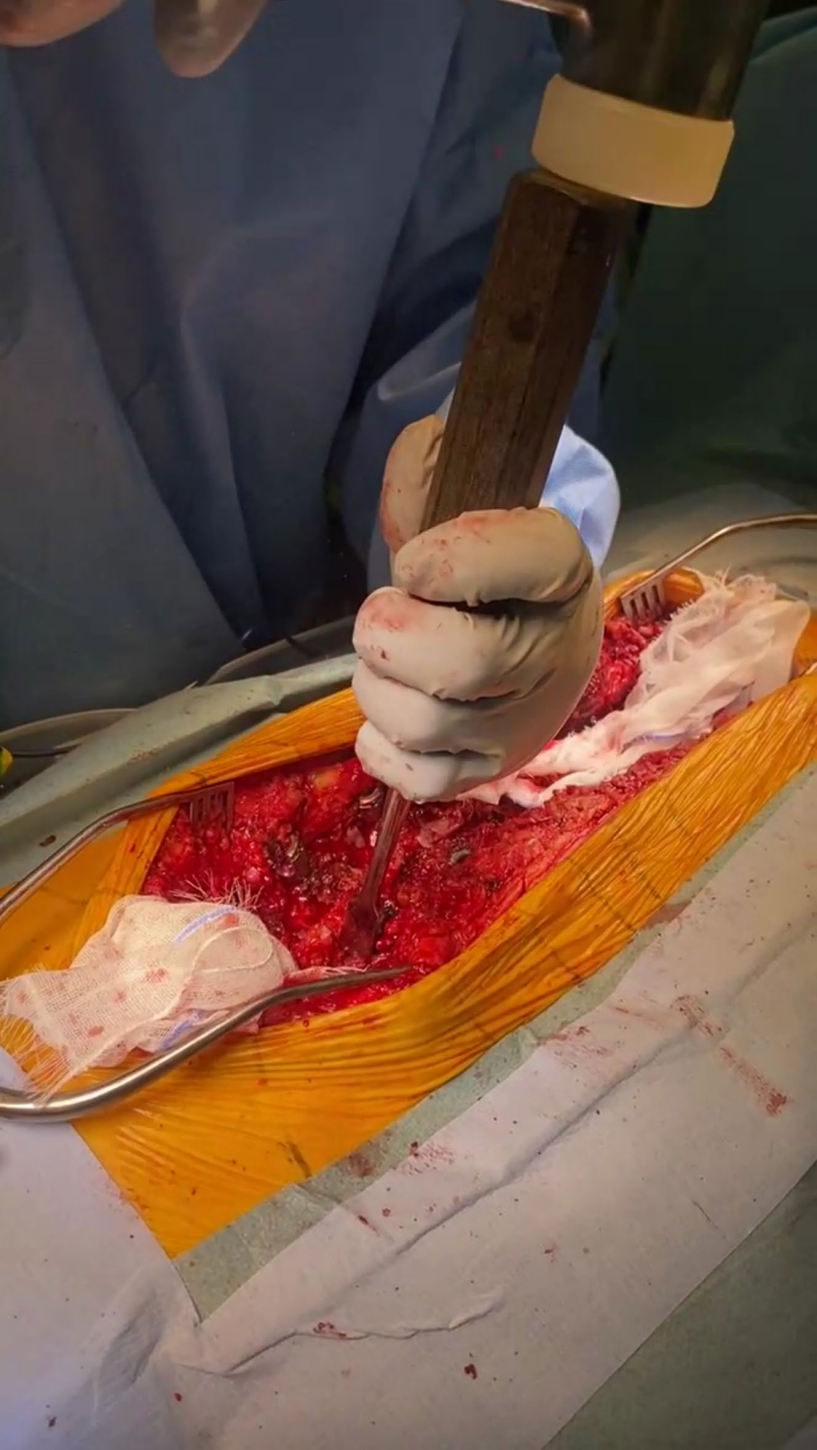
Performing a laminectomy with a chisel. The surgeon performed laminectomy or facetectomy using a hammer-and-chisel technique.

This study was approved by our institution’s ethics committee. This was a retrospective diagnostic accuracy study conducted in accordance with the Standards for Reporting Studies of Diagnostic accuracy for artificial intelligence (STARD-AI) guidelines^18^. Eligibility criteria were consecutive patients among those for whom intraoperative audio–video recordings were successfully obtained, who underwent lumbar or thoracic spine surgery with chisel-based bone resection at two spine centers between July 3, 2022, and September 12, 2024 (Fig. 1). All surgeries were performed by three experienced spine surgeons: Surgeon 1, Surgeon 2, and Surgeon 3, with 21, 19, and 14 years of experience, respectively. Percussion strikes with the chisel were recorded intraoperatively using an iPhone or iPad at a sampling frequency of 44.1 kHz. The chisels were made of stainless steel with resin handles (Fig. 2 and Supplementary video 1). In each percussion sequence, the chisel was tapped multiple times to gradually advance through the bone. When the chisel penetrated the cortical bone, the sound of the strike changed. In some procedures, surgeons avoided full cortical penetration by leaving a thin cortical layer and completing bone removal using levering or prying maneuvers. Because these techniques do not involve percussion-based cortical breakthrough or its characteristic acoustic change, such cases were excluded a priori from acoustic analysis.

**Fig. 3:**
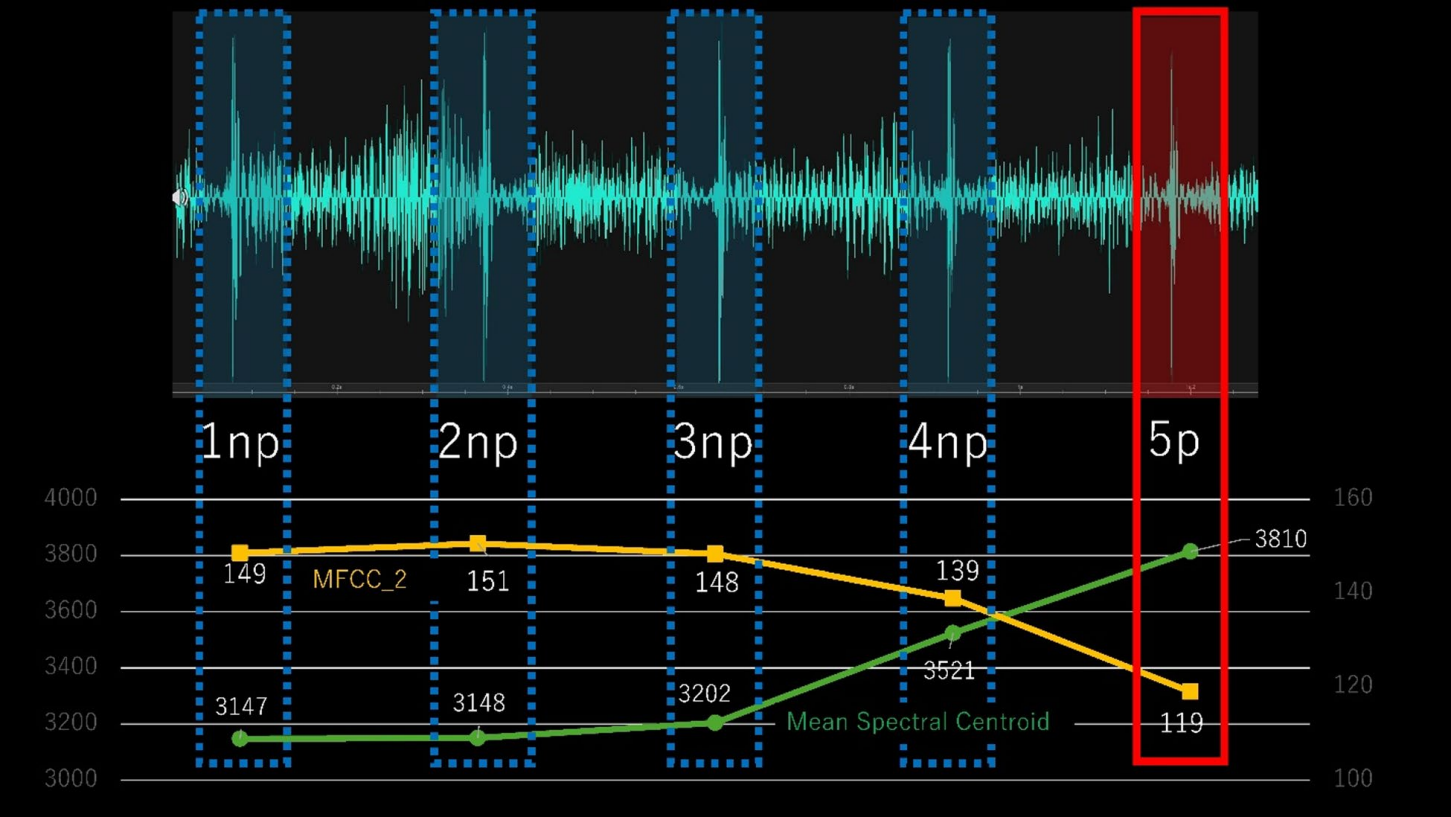
Example of annotation process for chisel percussion sounds. The upper panel shows a waveform segmented into individual strikes. Each strike was labeled as either non-penetration (np, blue dashed boxes) or penetration (p, red box) based on surgical video review and sound change. The lower panel illustrates two representative acoustic features (MFCC_2 in yellow and mean spectral centroid in green), highlighting that values typically shift when penetration occurs. These feature plots are shown to demonstrate the acoustic differences between np and p strikes.

Each strike was labeled as penetration (p) or non-penetration (np) (Fig. 3). Because no objective physical gold standard exists for identifying the exact moment of bone penetration, surgeon consensus review of the complete operative video served as the reference standard. Labeling was based on visual confirmation of cortical breakthrough and the transition to bone removal and/or the next surgical step, with acoustic changes considered only as supportive context. Initial labeling was performed by a spine surgeon with 6 years of experience and subsequently reviewed and confirmed by a senior spine surgeon with 21 years of experience, with final labels determined by consensus.

### Acoustic feature extraction

For each individual strike, 30 base acoustic features were extracted, including measures of energy, frequency distribution, mel-frequency cepstral coefficients (MFCC), and spectral patterns (Table 2). To better capture relative changes and scaling effects across consecutive strikes, additional derived features were computed, including differences, ratios, and log-transformed differences between strikes (Supplementary file 1).

**Fig. 4:**
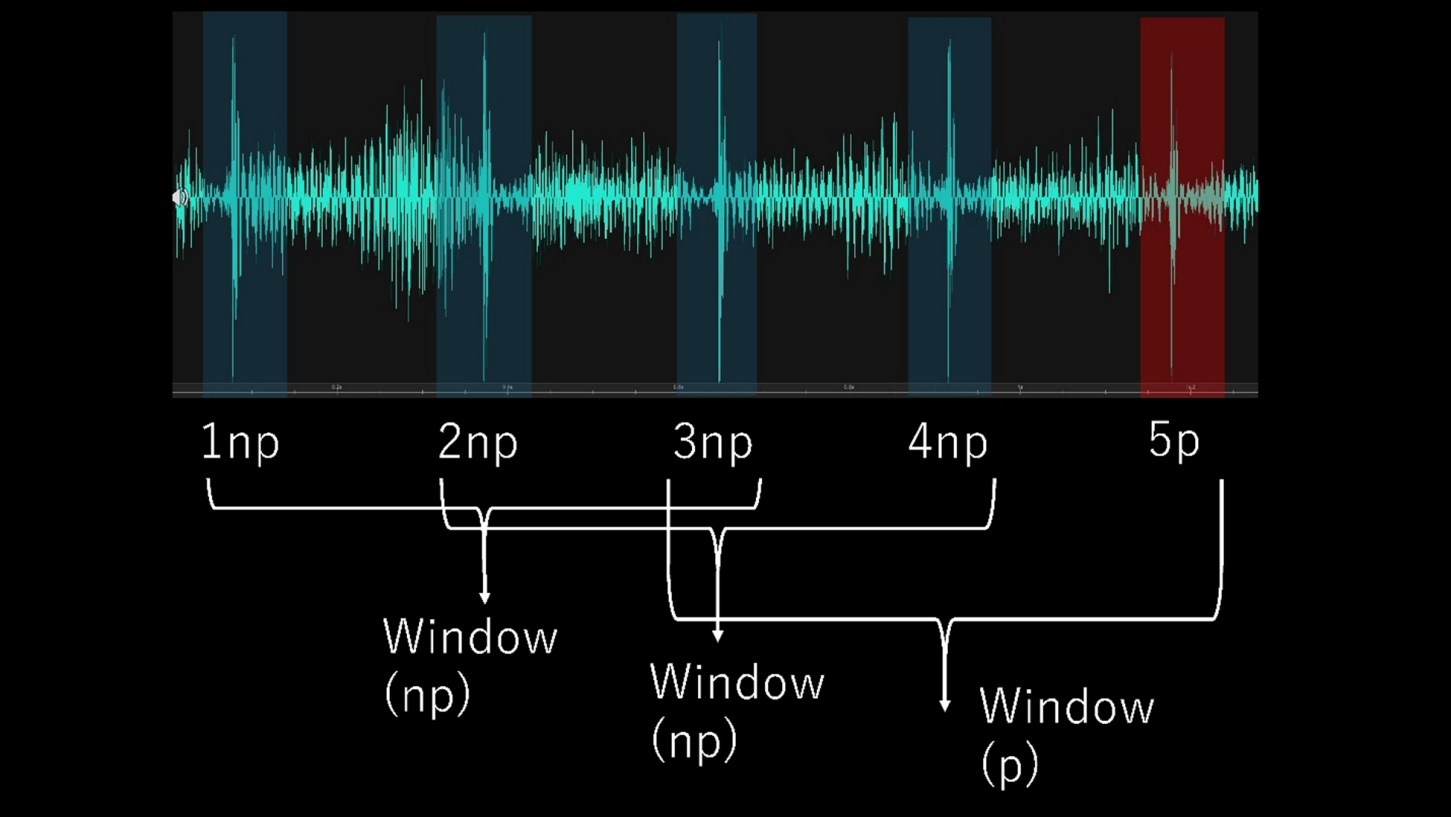
Construction of 3-hit windows for acoustic analysis. To capture temporal changes, consecutive strikes were grouped into 3-hit windows with a stride of 1. Each window inherited the label of the final strike within the sequence. For example, windows ending in 2np, 3np, and 4np were labeled as non-penetration, whereas the window ending in 5p was labeled as penetration.

A sliding window of three consecutive strikes (3-hit window, stride = 1) was then constructed (Fig. 4). For each 3-hit window, acoustic features were expanded to capture both the properties of individual strikes and the changes that occur between consecutive strikes. This process yielded a total of 570 features per window, enabling the model to learn not only how each strike sounds in isolation but also how the acoustic pattern evolves as the chisel approaches bone penetration.

### AI model development

A machine learning classifier was developed using LightGBM, trained on an expanded feature set comprising 570 features per 3-hit window. Data splitting was performed at the recording level to prevent information leakage between training and test sets.

### Benchmark comparison with a CNN-LSTM model

In response to reviewer comments, we additionally benchmarked the proposed feature-engineered LightGBM model against a representative convolutional neural network–long short-term memory (CNN-LSTM) model. For this benchmark, each 3-hit window was represented as a sequential input composed of the 30 base acoustic features extracted from each of the three consecutive strikes (input shape: 3 × 30). We intentionally used only the per-strike base features, rather than the expanded 570 hand-engineered window-level features, so that the CNN-LSTM could learn temporal relationships directly from minimally processed sequential inputs. The CNN-LSTM architecture consisted of a one-dimensional convolutional layer for local temporal feature extraction followed by an LSTM layer for sequential modeling and a final fully connected output layer for binary classification. Model development and evaluation were performed using the same recording-level data split, grouped 5-fold cross-validation, operating-threshold selection strategy, and independent hold-out test protocol as used for the proposed LightGBM model. Hyperparameters were selected based on cross-validation performance, with PR-AUC as the primary metric.

### Model selection and hyperparameter optimization

Model selection and hyperparameter optimization were conducted using 5-fold cross-validation on the training set, with grouping by recording identifier to ensure that all windows from a given recording session appeared exclusively in either the training or validation fold. The primary evaluation metric was the area under the precision–recall curve (PR-AUC), selected for its superior sensitivity to model performance on the minority (positive) class in imbalanced datasets. Model performance was summarized as mean values. 95% confidence intervals (CIs) for PR-AUC and ROC-AUC were estimated from fold-wise values using the t distribution. Secondary metrics included the area under the receiver operating characteristic curve (ROC-AUC) and threshold-dependent measures (accuracy, F1-score, precision, recall) computed at an optimized threshold that achieves sensitivity (recall) ≥ 80%. Early stopping (patience = 100 rounds) based on validation set PR-AUC was applied during cross-validation to identify the optimal number of boosting iterations and prevent overfitting.

### Operating threshold determination

The operating threshold for binary classification was determined using a clinically-driven approach prioritizing sensitivity. Given the importance of minimizing false negatives (missed bone breakthrough events) to prevent inadvertent injury to underlying structures, we targeted a minimum sensitivity of 80% based on clinical safety requirements. Using out-of-fold (OOF) predictions aggregated across all cross-validation folds, we analyzed the precision–recall curve and selected the highest probability threshold that achieved sensitivity (recall) ≥ 80%.

### Model training and evaluation

The final model was trained on 154 recordings using optimal hyperparameters identified during cross-validation, with 18 recordings reserved for internal validation. Training iterations were fixed to the mean optimal count across folds to ensure consistency with cross-validation results. To maintain consistency with cross-validation results, the model was trained for a fixed number of iterations equal to the mean optimal iteration count across folds. The internal validation set was used for monitoring purposes only and did not influence the model training process or stopping criteria. The final model was evaluated on the independent hold-out test set (20 recordings, 102 windows) using both threshold-independent metrics (PR-AUC, ROC-AUC) and threshold-dependent classification performance at the predetermined operating threshold.

For the independent hold-out test set, 95% confidence intervals (CIs) for ROC-AUC and PR-AUC were estimated using percentile-based cluster bootstrap resampling at the recording level (2,000 replicates) to account for within-recording correlation among overlapping 3-hit windows. In each bootstrap replicate, recordings were sampled with replacement, and all windows belonging to the sampled recordings were included in the resampled test set. Model performance was reported separately for cross-validation results reflecting training-set generalization, independent test-set performance at the clinically optimized operating threshold, and threshold sensitivity analysis showing performance trade-offs across multiple operating points.

### Statistical analysis

Univariate comparisons of individual acoustic features between penetration (p) and non-penetration (np) strikes were performed using the Mann–Whitney U test, given the non-normal distribution of the data.

A significance threshold of *p* < 0.05 (two-sided) was applied. All analyses were conducted using Python 3.10.14 with LightGBM 4.5.0, scikit-learn 1.5.2, pandas 2.2.2, and NumPy 2.1.3 (Supplementary file 2).

## Results

### Demographics

A total of 37 patients were included (mean age 61 ± 26 years, 25 with lumbar spinal stenosis and 12 with spinal deformity). From these patients, 739 surgical video clips were obtained. Among them, 192 audio records containing penetration sounds were extracted (Fig. 1). Each audio record contained an average of 6 ± 2 strikes (range 3–15), resulting in a total of 1236 labeled strikes: 215 penetration and 1021 non-penetration. The distribution of recordings across surgeons and dataset splits is summarized in Table 1.

**Table 1:** Recording-level distribution across surgeons and dataset splits.

Surgeon	Train (%)	Internal (%)	Test (%)	Total
Surgeon 1	112 (82.4)	14 (10.3)	10 (7.4)	136
Surgeon 2	24 (80.0)	2 (6.7)	4 (13.3)	30
Surgeon 3	18 (69.2)	2 (7.7)	6 (23.1)	26
Total	154 (80.2)	18 (9.4)	20 (10.4)	192

Data were split at the recording level. The numbers in parentheses indicate the percentage within each surgeon.

### Single-strike features

Comparison of acoustic features between penetration (*n* = 215) and non-penetration (*n* = 1021) strikes revealed significant differences in multiple spectral characteristics, including MFCC_2, MFCC_3, MFCC_6, MFCC_1, and MFCC_12, mean zero-crossing rate, mean spectral centroid, spectral roll-off, spectral flatness, and spectral contrast (Table 2). Most of these features represent tonal brightness or sharpness of the sound.

**Table 2:** Univariate analysis of 30 basic acoustic features.

Feature	Non-penetration	Penetration	*p*-value
MFCC_2	87.6 ± 34.8	68.4 ± 34.5	< 0.001
Mean Zero Crossing Rate	0.055 ± 0.02	0.051 ± 0.03	< 0.001
Mean Spectral Centroid (Hz)	5342 ± 1381	5870 ± 1395	< 0.001
MFCC_3	−53.1 ± 21.8	−61.1 ± 24.1	< 0.001
Contrast_7 (dB)	28.8 ± 4.5	30.3 ± 3.8	< 0.001
MFCC_6	17.1 ± 15	21.7 ± 13.3	< 0.001
Mean Amplitude	0.12 ± 0.05	0.11 ± 0.05	< 0.001
Mean Spectral Roll-off (Hz, 85%)	10,099 ± 2825	10,805 ± 2810	0.002
Contrast_1 (dB)	10.9 ± 4.4	9.9 ± 3.9	0.002
Mean Spectral Flatness	0.03 ± 0.03	0.02 ± 0.04	0.006
MFCC_1	−71.6 ± 35.2	−77.5 ± 36.3	0.007
MFCC_12	−4.7 ± 14.3	−2.4 ± 13.4	0.035
MFCC_8	22.1 ± 12.6	24.3 ± 13.3	0.059
Energy	25.1 ± 20.5	22.8 ± 17.1	0.084
MFCC_13	−11.2 ± 11.9	−10 ± 11.3	0.12
Contrast_4 (dB)	11 ± 3.3	11.2 ± 2.9	0.15
Duration (s)	0.02 ± 0.1	0.02 ± 0.01	0.18
Contrast_2 (dB)	4.6 ± 2.7	4.8 ± 2.9	0.20
MFCC_7	−14.8 ± 13.9	−13.7 ± 13.7	0.23
Mean RMS	0.1 ± 0.03	0.1 ± 0.04	0.24
Mean Spectral Bandwidth (Hz)	4295 ± 809	4360 ± 823	0.27
MFCC_5	−20.2 ± 17.8	−18.8 ± 16.5	0.39
MFCC_11	−7.1 ± 11.3	−8 ± 11.2	0.43
MFCC_9	−16.2 ± 12.3	−17.2 ± 11	0.44
Contrast_5 (dB)	12.4 ± 2.8	12.6 ± 2.6	0.48
Peaks Detected	1 ± 0.4	1 ± 0	0.65
Contrast_3 (dB)	8.4 ± 3.2	8.5 ± 3.4	0.79
MFCC_10	11.5 ± 11.8	11.5 ± 12	0.79
MFCC_4	32.1 ± 20.5	32 ± 21.1	0.81
Contrast_6 (dB)	14.8 ± 2.3	14.8 ± 2.3	0.89

The rows are sorted in ascending order of p-values from top to bottom.

*MFCC* mel-frequency cepstral coefficients.

### Three-strike windows

From the 1236 strikes, 852 three-strike windows were generated (215 penetration windows, 637 non-penetration windows; 25% vs. 75%) (Fig. 4). Among these, 20 audio records containing 102 windows (29 penetration windows, 73 non-penetration windows; 28% vs. 72%) were reserved as an independent test set. The remaining 172 audio records (750 windows: 186 penetration windows, 564 non-penetration windows; 25% vs. 75%) were further split into an internal training subset (154 recordings) and an internal validation subset (18 recordings).

### Model performance

Using the optimal hyperparameter configuration, the model demonstrated stable performance across 5-fold cross-validation, achieving a mean PR-AUC of 0.645 (95% CI, 0.546–0.744) and a mean ROC-AUC of 0.823 (95% CI, 0.754–0.891) (Table 3). Early stopping identified an average optimal training length of 106 iterations. Based on out-of-fold predictions, an operating threshold of 0.2305 was selected to achieve the prespecified target sensitivity of 0.80 on the training set, corresponding to an accuracy of 0.807, F1-score of 0.551, specificity of 0.635, and precision of 0.420. When evaluated on the independent hold-out test set, the model maintained robust discrimination, yielding a PR-AUC of 0.604 (95% CI, 0.463–0.818) and a ROC-AUC of 0.838 (95% CI, 0.740–0.925).

**Table 3:** Model performance of LightGBM at an operating threshold (0.2305).

	ROC-AUC	PR-AUC	Sensitivity (Recall)	Precision (Positive predictive value)	Accuracy	Specificity	F1-score	Threshold
Cross- validation (5-fold)	0.823 [0.754–0.891]	0.645 [0.546–0.744]	0.801	0.420	0.807	0.635	0.551	0.2305
Hold-out test	0.838 [0.740–0.925]	0.604 [0.463–0.818]	0.655	0.679	0.814	0.877	0.667	0.2305

*ROC-AUC* receiver operating characteristic curve – area under the curve, *PR-AUC* precision–recall – area under the curve.

Values in brackets indicate the 95% confidence interval.

### Benchmark comparison with a CNN-LSTM model

As an additional benchmark analysis, we compared the proposed method with a representative CNN-LSTM model using the same recording-level split and evaluation framework. The CNN-LSTM achieved a mean cross-validation PR-AUC of 0.589 and a hold-out test PR-AUC/ROC-AUC of 0.513/0.725, whereas the proposed LightGBM model achieved 0.645 in cross-validation PR-AUC and 0.604/0.838 on the hold-out test set, indicating better discrimination of the proposed approach in the current dataset. The detailed architecture and performance of the CNN-LSTM benchmark model are provided in the Supplementary file 4 and Supplementary Table 4.

### Threshold sensitivity analysis

Using the pre-specified operating threshold derived from out-of-fold predictions (0.2305), test-set performance was sensitivity 0.655, specificity 0.877, and precision 0.679 (Table 4). The corresponding confusion matrix for the independent test set is shown in Table 5. To illustrate the operating trade-offs, we additionally report test-set performance across alternative probability thresholds (Table 4). In an exploratory post-hoc assessment, we identified that a threshold of 0.15 would yield sensitivity ≥ 0.80 on this specific test set; at this threshold, sensitivity was 0.828, specificity 0.767, accuracy 0.784, and precision 0.585 (Tables 4 and 6).

**Table 4:** Threshold sensitivity analysis of test set.

Threshold	Sensitivity (recall)	Precision (Positive predictive value)	Accuracy	Specificity	F1-score
0.1	0.931	0.466	0.676	0.575	0.621
0.15*	0.828	0.585	0.784	0.767	0.686
0.20	0.724	0.656	0.814	0.849	0.688
0.2305†	0.655	0.679	0.814	0.877	0.667
0.25	0.621	0.692	0.814	0.890	0.655
0.30	0.586	0.680	0.804	0.890	0.628

*Exploratory post-hoc threshold yielding sensitivity ≥ 0.80 on the test set.

†Training-derived threshold from out of fold (OOF) predictions.

**Table 5:** Confusion matrix on the independent test set at the pre-specified operating threshold of 0.2305.

	AI prediction		
	Penetration	Non-penetration	Total
**Reference standard**			
Penetration	19 (TP)	10 (FN)	29
Non-penetration	9 (FP)	64 (TN)	73
Total	28	74	102

*TP* true positive, *FN* false negative, *FP* false positive, *TN* true negative.

Accuracy = 83/102 = 0.814, Recall (Sensitivity) = 19/29 = 0.655, Specificity = 64/73 = 0.877, Precision (Positive predictive value) = 19/28 = 0.679, F1-score = 0.667.

These values correspond to the threshold-dependent test-set performance reported at the pre-specified operating threshold.

**Table 6:** Confusion matrix on the independent test set at the threshold of 0.15.

	AI prediction		
	Penetration	Non-penetration	Total
**Reference standard**			
Penetration	24 (TP)	5 (FN)	29
Non-penetration	17 (FP)	56 (TN)	73
Total	41	61	102

*TP* true positive, *FN* false negative, *FP* false positive, *TN* true negative.

Accuracy = 80/102 = 0.784, Recall (Sensitivity) = 24/29 = 0.828, Specificity = 56/73 = 0.767, Precision (Positive predictive value) = 24/41 = 0.585, F1-score = 0.686.

### Feature importance

Feature importance analysis identified the most influential predictors as temporal dynamics of acoustic features across strikes (Table 7 and Supplementary file 3). The top-ranked feature was MFCC_3__slope3, representing the slope of the 3rd mel-frequency cepstral coefficient across the three-strike window. This was followed by MFCC_2__slope3 and MFCC_2__range3. Other highly ranked features included Contrast_7 (dB)__t3, MFCC_3__r21, and mean Zero Crossing Rate____std3.

**Table 7:** Top 10 feature importances of the model.

Ranking	Feature	Importance
1	MFCC_3__slope3	1294
2	MFCC_2__slope3	501
3	MFCC_2__range3	310
4	Contrast_7 (dB)__t3	280
5	MFCC_3__r21	261
6	Mean Zero Crossing Rate__std3	176
7	MFCC_10__range3	146
8	MFCC_5__t1	144
9	Contrast_1 (dB)__median3	136
10	MFCC_3__d21	134

## Discussion

In this study, we developed and validated an AI model capable of detecting bone penetration during spinal decompression surgery by analyzing chisel percussion sounds. The model achieved a PR-AUC of 0.645 in cross-validation and 0.604 in an independent test set, with ROC-AUC values consistently above 0.80. Given that the prevalence of penetration events in our dataset was relatively low (≈ 17%), these PR-AUC values represent meaningful discriminative performance well above chance level, where a random classifier would be expected to achieve only ~ 0.17. These findings indicate that the approach generalizes reasonably well to unseen data despite the limited sample size^19,20^. Feature importance analysis further showed that the model relied primarily on changes, slopes, and ranges of acoustic features across sequential strikes rather than on static single-strike values, supporting the importance of temporal information for detecting penetration.

### Study novelty

To our knowledge, this is the first report to apply machine learning to intraoperative percussion sounds in spinal surgery. Previous studies in medicine have mainly focused on respiratory sounds^14^, cardiac auscultation^15,21^, or industrial quality-control tasks^13^, rather than surgical sound analysis. Although experienced surgeons have long used subtle acoustic cues during procedures such as laminectomy or arthroplasty, these judgments have remained subjective and dependent on years of training^1,2,5,6^. Our study demonstrates that such cues can be objectively captured, quantified, and classified using AI. By using three-strike windows, the proposed approach was designed to capture evolving acoustic patterns rather than isolated sounds, which may better reflect how penetration is recognized in practice. This methodological perspective may help establish surgical acoustics as a new application area within the broader field of Physical AI.

### Clinical relevance

The clinical implications of this work are considerable. First, objective recognition of bone penetration may support less experienced surgeons during spinal decompression by providing real-time feedback and helping them learn sensory cues that are otherwise acquired through prolonged practice. Such a system may also reduce inter-surgeon variability and improve safety in procedures requiring precise bony removal. For example, when a junior surgeon performs bone cutting, an experienced supervisor may recognize from sound alone that penetration has already occurred and intervene to prevent excessive chiseling. By making this judgment explicit and accessible, the proposed model may help enhance both safety and training.

Our results also show that different probability thresholds produce predictable trade-offs between sensitivity and specificity, suggesting that the model could be tuned according to the clinical context. Second, as surgical robotics continues to advance^17^, integration of acoustic AI could provide robots with an additional sensory modality, complementing force or visual feedback and enabling safer autonomous or semi-autonomous bone cutting^22,23^. In this sense, our work represents an early step toward Surgeon-assist Physical AI (SPAI), an AI framework intended to emulate and augment surgeons’ intraoperative sensory judgment.

### Why the sound changes

The acoustic change that occurs when the chisel penetrates bone can be explained by basic principles of material vibration^6,24–26^. When a chisel strikes cortical bone, the sound is generated by vibration of both the chisel and the bone structure. Before penetration, the chisel is resisted by solid bone, producing a duller and more uniform tone. Once penetration occurs, local structural integrity is lost, resulting in a sharper, higher-pitched sound^2^. This is analogous to the difference between striking a solid wall and a hollow structure: the resonance shifts, and the resulting sound becomes brighter and more distinct.

### Acoustic features and their interpretation

The model’s reliance on features such as mel-frequency cepstral coefficients (MFCCs), zero-crossing rate, and spectral contrast reflects the acoustic properties that changed most prominently during penetration^27,28^. Notably, the top predictors were predominantly measures of temporal dynamics, including slopes, ranges, and ratios across consecutive strikes, indicating that the model captured not only the tonal quality of each strike but also its progression over time.

MFCCs are widely used in speech and audio recognition to represent the timbre or tonal quality of a sound. In our analysis, slope- and range-based MFCC features, such as MFCC_3__slope3, MFCC_2__slope3, and MFCC_2__range3, ranked among the most important predictors, suggesting that penetration was associated with systematic shifts in tonal patterns across the three-strike window. Taken together, these findings provide an objective explanation for the acoustic changes that surgeons subjectively recognize at the time of penetration.

#### Limitations

This study has some limitations. First, the dataset was relatively small and derived from procedures performed by experienced spine surgeons. Although the number of individual strikes was sufficient for feature extraction, the diversity of surgical contexts was limited.

Second, the present approach relied on manually segmented individual strikes and the construction of fixed three-strike windows. While effective as a proof of concept, this design does not yet analyze the full continuous acoustic stream as it would occur intraoperatively.

Third, although we added a benchmark comparison with a representative CNN-LSTM model in response to reviewer comments, this benchmark did not outperform the proposed LightGBM model in the current dataset. One possible explanation is that the available dataset may still have been limited for deep sequential modeling. In addition, although raw-audio input to deep learning models may capture information beyond handcrafted acoustic features, we did not adopt this strategy in the present comparison because it would have substantially changed the input representation and reduced the comparability of the benchmark with our feature-engineered approach. Future studies with larger datasets should evaluate raw-audio and attention-based deep learning models to further clarify the role of end-to-end sequential modeling in intraoperative acoustic analysis.

## Conclusion

In summary, this study demonstrates the feasibility of using machine learning to recognize bone penetration during spinal decompression by analyzing acoustic signals from chisel strikes. This approach could enhance surgical training, provide real-time decision support for less experienced surgeons, and ultimately serve as a foundation for multimodal feedback systems in robotic or computer-assisted surgery.

## Supplementary Information

Below is the link to the electronic supplementary material.

Supplementary Material 1

Supplementary Material 2

Supplementary Material 3

Supplementary Material 4

Supplementary Material 5

Supplementary Material 6

## Data Availability

All data generated or analyzed during this study are available upon request from the corresponding author.
