# Supplementary material for: Acoustic cues into a surgeon-assist physical AI for detecting bone penetration during spinal surgery
Source: Sci Rep. 2026 Apr 19;16:18113. doi: 10.1038/s41598-026-48857-w (PMC13254284; doi:10.1038/s41598-026-48857-w)
Supplement: Supplementary file 2 — Supplementary Material 2 [file 41598_2026_48857_MOESM2_ESM.docx]

**Supplementary Material:**

**Feature Engineering and Expansion**

For each 3-hit window, we began with 30 base acoustic features (energy, amplitude, zero-crossing rate, spectral descriptors, 7 bands of spectral contrast, and 13 mel-frequency cepstral coefficients [MFCCs]). To capture both the absolute properties of each strike and the temporal evolution across consecutive strikes, we systematically expanded these base features into 19 derived representations:

**1, 2, and 3:** t1, t2, t3 – raw feature values for the 1st, 2nd, and 3rd strikes

**4, and 5**: d21, d32 – differences between consecutive strikes (2nd–1st, 3rd–2nd)

**6:** mean3 – mean across the three strikes

**7:** std3 – standard deviation across the three strikes

**8:** median3 – median across the three strikes

**9:** range3 – range (max–min) across the three strikes

**10, and 11:** r21, r32 – ratios between consecutive strikes (2nd/1st, 3rd/2nd)

**12, and 13:** pct21, pct32 – percent change between consecutive strikes

**14, 15, and 16:** log_t1, log_t2, log_t3 – log-transformed raw values (stability for skewed distributions)

**17, and 18:** logd21, logd32 – log-transformed differences between consecutive strikes

**19:** slope3 – linear slope fitted across the three strike values

This systematic expansion produced a total of 570 features per window (30 base × 19 transforms). While many of these derived values are correlated, they provide complementary perspectives:

absolute values capture the instantaneous acoustic property of each strike, differences, ratios, and percent changes highlight dynamic changes between strikes, log transforms reduce skewness and emphasize relative magnitude changes, and slope3 quantifies the overall trajectory across the three strikes.

| **Supplementary Table 1. Naming conventions of acoustic features per 3-hit window.** | | | |
| --- | --- | --- | --- |
| **Suffix** | **Meaning** | **Example** | **Description** |
| __t1 | Value at the 1st strike | MFCC_2__t1 | Feature value at the first strike of the 3-hit window |
| __t2 | Value at the 2nd strike | Spectral Centroid__t2 | Feature value at the second strike |
| __t3 | Value at the 3rd strike | Contrast_7 (dB)__t3 | Feature value at the third strike |
| __d21 | Difference: 2nd − 1st strike | MFCC_3__d21 | Captures change from 1st to 2nd strike |
| __d32 | Difference: 3rd − 2nd strike | Mean Zero Crossing Rate__d32 | Captures change from 2nd to 3rd strike |
| __mean3 | Mean across 3 strikes | Spectral Flatness__mean3 | Overall tendency or stability |
| __std3 | Standard deviation across 3 strikes | MFCC_4__std3 | Variability across 3 strikes |
| __median3 | Median across 3 strikes | MFCC_5__median3 | Robust central tendency |
| __range3 | Range (max − min) across 3 strikes | MFCC_6__range3 | Spread of values |
| __r21 | Ratio (2nd / 1st) | MFCC_7__r21 | Relative change between 1st and 2nd |
| __r32 | Ratio (3rd / 2nd) | MFCC_8__r32 | Relative change between 2nd and 3rd |
| __pct21 | Percent change wrt 1st | MFCC_9__pct21 | (2nd − 1st)/\|1st\| |
| __pct32 | Percent change wrt 2nd | MFCC_10__pct32 | (3rd − 2nd)/\|2nd\| |
| __log_t1 | Log-transformed value at 1st strike | MFCC_11__log_t1 | Signed log to stabilize scale |
| __log_t2 | Log-transformed value at 2nd strike | MFCC_12__log_t2 | Signed log to stabilize scale |
| __log_t3 | Log-transformed value at 3rd strike | MFCC_13__log_t3 | Signed log to stabilize scale |
| __logd21 | Log-transformed difference (2nd − 1st) | MFCC_1__logd21 | Captures relative local change |
| __logd32 | Log-transformed difference (3rd − 2nd) | Spectral Centroid__logd32 | Captures relative later change |
| __slope3 | Linear slope across 3 strikes | MFCC_2__slope3 | Trend across 3 strikes |

| **Supplementary Table 2. 570 features per window.** | | | |
| --- | --- | --- | --- |
| Number | Base Feature | Variant | Final Feature Name |
| 1 | Contrast_1 (dB) | d21 | Contrast_1 (dB)__d21 |
| 2 | Contrast_1 (dB) | d32 | Contrast_1 (dB)__d32 |
| 3 | Contrast_1 (dB) | log_t1 | Contrast_1 (dB)__log_t1 |
| 4 | Contrast_1 (dB) | log_t2 | Contrast_1 (dB)__log_t2 |
| 5 | Contrast_1 (dB) | log_t3 | Contrast_1 (dB)__log_t3 |
| 6 | Contrast_1 (dB) | logd21 | Contrast_1 (dB)__logd21 |
| 7 | Contrast_1 (dB) | logd32 | Contrast_1 (dB)__logd32 |
| 8 | Contrast_1 (dB) | mean3 | Contrast_1 (dB)__mean3 |
| 9 | Contrast_1 (dB) | median3 | Contrast_1 (dB)__median3 |
| 10 | Contrast_1 (dB) | pct21 | Contrast_1 (dB)__pct21 |
| 11 | Contrast_1 (dB) | pct32 | Contrast_1 (dB)__pct32 |
| 12 | Contrast_1 (dB) | r21 | Contrast_1 (dB)__r21 |
| 13 | Contrast_1 (dB) | r32 | Contrast_1 (dB)__r32 |
| 14 | Contrast_1 (dB) | range3 | Contrast_1 (dB)__range3 |
| 15 | Contrast_1 (dB) | slope3 | Contrast_1 (dB)__slope3 |
| 16 | Contrast_1 (dB) | std3 | Contrast_1 (dB)__std3 |
| 17 | Contrast_1 (dB) | t1 | Contrast_1 (dB)__t1 |
| 18 | Contrast_1 (dB) | t2 | Contrast_1 (dB)__t2 |
| 19 | Contrast_1 (dB) | t3 | Contrast_1 (dB)__t3 |
| 20 | Contrast_2 (dB) | d21 | Contrast_2 (dB)__d21 |
| 21 | Contrast_2 (dB) | d32 | Contrast_2 (dB)__d32 |
| 22 | Contrast_2 (dB) | log_t1 | Contrast_2 (dB)__log_t1 |
| 23 | Contrast_2 (dB) | log_t2 | Contrast_2 (dB)__log_t2 |
| 24 | Contrast_2 (dB) | log_t3 | Contrast_2 (dB)__log_t3 |
| 25 | Contrast_2 (dB) | logd21 | Contrast_2 (dB)__logd21 |
| 26 | Contrast_2 (dB) | logd32 | Contrast_2 (dB)__logd32 |
| 27 | Contrast_2 (dB) | mean3 | Contrast_2 (dB)__mean3 |
| 28 | Contrast_2 (dB) | median3 | Contrast_2 (dB)__median3 |
| 29 | Contrast_2 (dB) | pct21 | Contrast_2 (dB)__pct21 |
| 30 | Contrast_2 (dB) | pct32 | Contrast_2 (dB)__pct32 |
| 31 | Contrast_2 (dB) | r21 | Contrast_2 (dB)__r21 |
| 32 | Contrast_2 (dB) | r32 | Contrast_2 (dB)__r32 |
| 33 | Contrast_2 (dB) | range3 | Contrast_2 (dB)__range3 |
| 34 | Contrast_2 (dB) | slope3 | Contrast_2 (dB)__slope3 |
| 35 | Contrast_2 (dB) | std3 | Contrast_2 (dB)__std3 |
| 36 | Contrast_2 (dB) | t1 | Contrast_2 (dB)__t1 |
| 37 | Contrast_2 (dB) | t2 | Contrast_2 (dB)__t2 |
| 38 | Contrast_2 (dB) | t3 | Contrast_2 (dB)__t3 |
| 39 | Contrast_3 (dB) | d21 | Contrast_3 (dB)__d21 |
| 40 | Contrast_3 (dB) | d32 | Contrast_3 (dB)__d32 |
| 41 | Contrast_3 (dB) | log_t1 | Contrast_3 (dB)__log_t1 |
| 42 | Contrast_3 (dB) | log_t2 | Contrast_3 (dB)__log_t2 |
| 43 | Contrast_3 (dB) | log_t3 | Contrast_3 (dB)__log_t3 |
| 44 | Contrast_3 (dB) | logd21 | Contrast_3 (dB)__logd21 |
| 45 | Contrast_3 (dB) | logd32 | Contrast_3 (dB)__logd32 |
| 46 | Contrast_3 (dB) | mean3 | Contrast_3 (dB)__mean3 |
| 47 | Contrast_3 (dB) | median3 | Contrast_3 (dB)__median3 |
| 48 | Contrast_3 (dB) | pct21 | Contrast_3 (dB)__pct21 |
| 49 | Contrast_3 (dB) | pct32 | Contrast_3 (dB)__pct32 |
| 50 | Contrast_3 (dB) | r21 | Contrast_3 (dB)__r21 |
| 51 | Contrast_3 (dB) | r32 | Contrast_3 (dB)__r32 |
| 52 | Contrast_3 (dB) | range3 | Contrast_3 (dB)__range3 |
| 53 | Contrast_3 (dB) | slope3 | Contrast_3 (dB)__slope3 |
| 54 | Contrast_3 (dB) | std3 | Contrast_3 (dB)__std3 |
| 55 | Contrast_3 (dB) | t1 | Contrast_3 (dB)__t1 |
| 56 | Contrast_3 (dB) | t2 | Contrast_3 (dB)__t2 |
| 57 | Contrast_3 (dB) | t3 | Contrast_3 (dB)__t3 |
| 58 | Contrast_4 (dB) | d21 | Contrast_4 (dB)__d21 |
| 59 | Contrast_4 (dB) | d32 | Contrast_4 (dB)__d32 |
| 60 | Contrast_4 (dB) | log_t1 | Contrast_4 (dB)__log_t1 |
| 61 | Contrast_4 (dB) | log_t2 | Contrast_4 (dB)__log_t2 |
| 62 | Contrast_4 (dB) | log_t3 | Contrast_4 (dB)__log_t3 |
| 63 | Contrast_4 (dB) | logd21 | Contrast_4 (dB)__logd21 |
| 64 | Contrast_4 (dB) | logd32 | Contrast_4 (dB)__logd32 |
| 65 | Contrast_4 (dB) | mean3 | Contrast_4 (dB)__mean3 |
| 66 | Contrast_4 (dB) | median3 | Contrast_4 (dB)__median3 |
| 67 | Contrast_4 (dB) | pct21 | Contrast_4 (dB)__pct21 |
| 68 | Contrast_4 (dB) | pct32 | Contrast_4 (dB)__pct32 |
| 69 | Contrast_4 (dB) | r21 | Contrast_4 (dB)__r21 |
| 70 | Contrast_4 (dB) | r32 | Contrast_4 (dB)__r32 |
| 71 | Contrast_4 (dB) | range3 | Contrast_4 (dB)__range3 |
| 72 | Contrast_4 (dB) | slope3 | Contrast_4 (dB)__slope3 |
| 73 | Contrast_4 (dB) | std3 | Contrast_4 (dB)__std3 |
| 74 | Contrast_4 (dB) | t1 | Contrast_4 (dB)__t1 |
| 75 | Contrast_4 (dB) | t2 | Contrast_4 (dB)__t2 |
| 76 | Contrast_4 (dB) | t3 | Contrast_4 (dB)__t3 |
| 77 | Contrast_5 (dB) | d21 | Contrast_5 (dB)__d21 |
| 78 | Contrast_5 (dB) | d32 | Contrast_5 (dB)__d32 |
| 79 | Contrast_5 (dB) | log_t1 | Contrast_5 (dB)__log_t1 |
| 80 | Contrast_5 (dB) | log_t2 | Contrast_5 (dB)__log_t2 |
| 81 | Contrast_5 (dB) | log_t3 | Contrast_5 (dB)__log_t3 |
| 82 | Contrast_5 (dB) | logd21 | Contrast_5 (dB)__logd21 |
| 83 | Contrast_5 (dB) | logd32 | Contrast_5 (dB)__logd32 |
| 84 | Contrast_5 (dB) | mean3 | Contrast_5 (dB)__mean3 |
| 85 | Contrast_5 (dB) | median3 | Contrast_5 (dB)__median3 |
| 86 | Contrast_5 (dB) | pct21 | Contrast_5 (dB)__pct21 |
| 87 | Contrast_5 (dB) | pct32 | Contrast_5 (dB)__pct32 |
| 88 | Contrast_5 (dB) | r21 | Contrast_5 (dB)__r21 |
| 89 | Contrast_5 (dB) | r32 | Contrast_5 (dB)__r32 |
| 90 | Contrast_5 (dB) | range3 | Contrast_5 (dB)__range3 |
| 91 | Contrast_5 (dB) | slope3 | Contrast_5 (dB)__slope3 |
| 92 | Contrast_5 (dB) | std3 | Contrast_5 (dB)__std3 |
| 93 | Contrast_5 (dB) | t1 | Contrast_5 (dB)__t1 |
| 94 | Contrast_5 (dB) | t2 | Contrast_5 (dB)__t2 |
| 95 | Contrast_5 (dB) | t3 | Contrast_5 (dB)__t3 |
| 96 | Contrast_6 (dB) | d21 | Contrast_6 (dB)__d21 |
| 97 | Contrast_6 (dB) | d32 | Contrast_6 (dB)__d32 |
| 98 | Contrast_6 (dB) | log_t1 | Contrast_6 (dB)__log_t1 |
| 99 | Contrast_6 (dB) | log_t2 | Contrast_6 (dB)__log_t2 |
| 100 | Contrast_6 (dB) | log_t3 | Contrast_6 (dB)__log_t3 |
| 101 | Contrast_6 (dB) | logd21 | Contrast_6 (dB)__logd21 |
| 102 | Contrast_6 (dB) | logd32 | Contrast_6 (dB)__logd32 |
| 103 | Contrast_6 (dB) | mean3 | Contrast_6 (dB)__mean3 |
| 104 | Contrast_6 (dB) | median3 | Contrast_6 (dB)__median3 |
| 105 | Contrast_6 (dB) | pct21 | Contrast_6 (dB)__pct21 |
| 106 | Contrast_6 (dB) | pct32 | Contrast_6 (dB)__pct32 |
| 107 | Contrast_6 (dB) | r21 | Contrast_6 (dB)__r21 |
| 108 | Contrast_6 (dB) | r32 | Contrast_6 (dB)__r32 |
| 109 | Contrast_6 (dB) | range3 | Contrast_6 (dB)__range3 |
| 110 | Contrast_6 (dB) | slope3 | Contrast_6 (dB)__slope3 |
| 111 | Contrast_6 (dB) | std3 | Contrast_6 (dB)__std3 |
| 112 | Contrast_6 (dB) | t1 | Contrast_6 (dB)__t1 |
| 113 | Contrast_6 (dB) | t2 | Contrast_6 (dB)__t2 |
| 114 | Contrast_6 (dB) | t3 | Contrast_6 (dB)__t3 |
| 115 | Contrast_7 (dB) | d21 | Contrast_7 (dB)__d21 |
| 116 | Contrast_7 (dB) | d32 | Contrast_7 (dB)__d32 |
| 117 | Contrast_7 (dB) | log_t1 | Contrast_7 (dB)__log_t1 |
| 118 | Contrast_7 (dB) | log_t2 | Contrast_7 (dB)__log_t2 |
| 119 | Contrast_7 (dB) | log_t3 | Contrast_7 (dB)__log_t3 |
| 120 | Contrast_7 (dB) | logd21 | Contrast_7 (dB)__logd21 |
| 121 | Contrast_7 (dB) | logd32 | Contrast_7 (dB)__logd32 |
| 122 | Contrast_7 (dB) | mean3 | Contrast_7 (dB)__mean3 |
| 123 | Contrast_7 (dB) | median3 | Contrast_7 (dB)__median3 |
| 124 | Contrast_7 (dB) | pct21 | Contrast_7 (dB)__pct21 |
| 125 | Contrast_7 (dB) | pct32 | Contrast_7 (dB)__pct32 |
| 126 | Contrast_7 (dB) | r21 | Contrast_7 (dB)__r21 |
| 127 | Contrast_7 (dB) | r32 | Contrast_7 (dB)__r32 |
| 128 | Contrast_7 (dB) | range3 | Contrast_7 (dB)__range3 |
| 129 | Contrast_7 (dB) | slope3 | Contrast_7 (dB)__slope3 |
| 130 | Contrast_7 (dB) | std3 | Contrast_7 (dB)__std3 |
| 131 | Contrast_7 (dB) | t1 | Contrast_7 (dB)__t1 |
| 132 | Contrast_7 (dB) | t2 | Contrast_7 (dB)__t2 |
| 133 | Contrast_7 (dB) | t3 | Contrast_7 (dB)__t3 |
| 134 | Duration (s) | d21 | Duration (s)__d21 |
| 135 | Duration (s) | d32 | Duration (s)__d32 |
| 136 | Duration (s) | log_t1 | Duration (s)__log_t1 |
| 137 | Duration (s) | log_t2 | Duration (s)__log_t2 |
| 138 | Duration (s) | log_t3 | Duration (s)__log_t3 |
| 139 | Duration (s) | logd21 | Duration (s)__logd21 |
| 140 | Duration (s) | logd32 | Duration (s)__logd32 |
| 141 | Duration (s) | mean3 | Duration (s)__mean3 |
| 142 | Duration (s) | median3 | Duration (s)__median3 |
| 143 | Duration (s) | pct21 | Duration (s)__pct21 |
| 144 | Duration (s) | pct32 | Duration (s)__pct32 |
| 145 | Duration (s) | r21 | Duration (s)__r21 |
| 146 | Duration (s) | r32 | Duration (s)__r32 |
| 147 | Duration (s) | range3 | Duration (s)__range3 |
| 148 | Duration (s) | slope3 | Duration (s)__slope3 |
| 149 | Duration (s) | std3 | Duration (s)__std3 |
| 150 | Duration (s) | t1 | Duration (s)__t1 |
| 151 | Duration (s) | t2 | Duration (s)__t2 |
| 152 | Duration (s) | t3 | Duration (s)__t3 |
| 153 | Energy | d21 | Energy__d21 |
| 154 | Energy | d32 | Energy__d32 |
| 155 | Energy | log_t1 | Energy__log_t1 |
| 156 | Energy | log_t2 | Energy__log_t2 |
| 157 | Energy | log_t3 | Energy__log_t3 |
| 158 | Energy | logd21 | Energy__logd21 |
| 159 | Energy | logd32 | Energy__logd32 |
| 160 | Energy | mean3 | Energy__mean3 |
| 161 | Energy | median3 | Energy__median3 |
| 162 | Energy | pct21 | Energy__pct21 |
| 163 | Energy | pct32 | Energy__pct32 |
| 164 | Energy | r21 | Energy__r21 |
| 165 | Energy | r32 | Energy__r32 |
| 166 | Energy | range3 | Energy__range3 |
| 167 | Energy | slope3 | Energy__slope3 |
| 168 | Energy | std3 | Energy__std3 |
| 169 | Energy | t1 | Energy__t1 |
| 170 | Energy | t2 | Energy__t2 |
| 171 | Energy | t3 | Energy__t3 |
| 172 | MFCC_1 | d21 | MFCC_1__d21 |
| 173 | MFCC_1 | d32 | MFCC_1__d32 |
| 174 | MFCC_1 | log_t1 | MFCC_1__log_t1 |
| 175 | MFCC_1 | log_t2 | MFCC_1__log_t2 |
| 176 | MFCC_1 | log_t3 | MFCC_1__log_t3 |
| 177 | MFCC_1 | logd21 | MFCC_1__logd21 |
| 178 | MFCC_1 | logd32 | MFCC_1__logd32 |
| 179 | MFCC_1 | mean3 | MFCC_1__mean3 |
| 180 | MFCC_1 | median3 | MFCC_1__median3 |
| 181 | MFCC_1 | pct21 | MFCC_1__pct21 |
| 182 | MFCC_1 | pct32 | MFCC_1__pct32 |
| 183 | MFCC_1 | r21 | MFCC_1__r21 |
| 184 | MFCC_1 | r32 | MFCC_1__r32 |
| 185 | MFCC_1 | range3 | MFCC_1__range3 |
| 186 | MFCC_1 | slope3 | MFCC_1__slope3 |
| 187 | MFCC_1 | std3 | MFCC_1__std3 |
| 188 | MFCC_1 | t1 | MFCC_1__t1 |
| 189 | MFCC_1 | t2 | MFCC_1__t2 |
| 190 | MFCC_1 | t3 | MFCC_1__t3 |
| 191 | MFCC_10 | d21 | MFCC_10__d21 |
| 192 | MFCC_10 | d32 | MFCC_10__d32 |
| 193 | MFCC_10 | log_t1 | MFCC_10__log_t1 |
| 194 | MFCC_10 | log_t2 | MFCC_10__log_t2 |
| 195 | MFCC_10 | log_t3 | MFCC_10__log_t3 |
| 196 | MFCC_10 | logd21 | MFCC_10__logd21 |
| 197 | MFCC_10 | logd32 | MFCC_10__logd32 |
| 198 | MFCC_10 | mean3 | MFCC_10__mean3 |
| 199 | MFCC_10 | median3 | MFCC_10__median3 |
| 200 | MFCC_10 | pct21 | MFCC_10__pct21 |
| 201 | MFCC_10 | pct32 | MFCC_10__pct32 |
| 202 | MFCC_10 | r21 | MFCC_10__r21 |
| 203 | MFCC_10 | r32 | MFCC_10__r32 |
| 204 | MFCC_10 | range3 | MFCC_10__range3 |
| 205 | MFCC_10 | slope3 | MFCC_10__slope3 |
| 206 | MFCC_10 | std3 | MFCC_10__std3 |
| 207 | MFCC_10 | t1 | MFCC_10__t1 |
| 208 | MFCC_10 | t2 | MFCC_10__t2 |
| 209 | MFCC_10 | t3 | MFCC_10__t3 |
| 210 | MFCC_11 | d21 | MFCC_11__d21 |
| 211 | MFCC_11 | d32 | MFCC_11__d32 |
| 212 | MFCC_11 | log_t1 | MFCC_11__log_t1 |
| 213 | MFCC_11 | log_t2 | MFCC_11__log_t2 |
| 214 | MFCC_11 | log_t3 | MFCC_11__log_t3 |
| 215 | MFCC_11 | logd21 | MFCC_11__logd21 |
| 216 | MFCC_11 | logd32 | MFCC_11__logd32 |
| 217 | MFCC_11 | mean3 | MFCC_11__mean3 |
| 218 | MFCC_11 | median3 | MFCC_11__median3 |
| 219 | MFCC_11 | pct21 | MFCC_11__pct21 |
| 220 | MFCC_11 | pct32 | MFCC_11__pct32 |
| 221 | MFCC_11 | r21 | MFCC_11__r21 |
| 222 | MFCC_11 | r32 | MFCC_11__r32 |
| 223 | MFCC_11 | range3 | MFCC_11__range3 |
| 224 | MFCC_11 | slope3 | MFCC_11__slope3 |
| 225 | MFCC_11 | std3 | MFCC_11__std3 |
| 226 | MFCC_11 | t1 | MFCC_11__t1 |
| 227 | MFCC_11 | t2 | MFCC_11__t2 |
| 228 | MFCC_11 | t3 | MFCC_11__t3 |
| 229 | MFCC_12 | d21 | MFCC_12__d21 |
| 230 | MFCC_12 | d32 | MFCC_12__d32 |
| 231 | MFCC_12 | log_t1 | MFCC_12__log_t1 |
| 232 | MFCC_12 | log_t2 | MFCC_12__log_t2 |
| 233 | MFCC_12 | log_t3 | MFCC_12__log_t3 |
| 234 | MFCC_12 | logd21 | MFCC_12__logd21 |
| 235 | MFCC_12 | logd32 | MFCC_12__logd32 |
| 236 | MFCC_12 | mean3 | MFCC_12__mean3 |
| 237 | MFCC_12 | median3 | MFCC_12__median3 |
| 238 | MFCC_12 | pct21 | MFCC_12__pct21 |
| 239 | MFCC_12 | pct32 | MFCC_12__pct32 |
| 240 | MFCC_12 | r21 | MFCC_12__r21 |
| 241 | MFCC_12 | r32 | MFCC_12__r32 |
| 242 | MFCC_12 | range3 | MFCC_12__range3 |
| 243 | MFCC_12 | slope3 | MFCC_12__slope3 |
| 244 | MFCC_12 | std3 | MFCC_12__std3 |
| 245 | MFCC_12 | t1 | MFCC_12__t1 |
| 246 | MFCC_12 | t2 | MFCC_12__t2 |
| 247 | MFCC_12 | t3 | MFCC_12__t3 |
| 248 | MFCC_13 | d21 | MFCC_13__d21 |
| 249 | MFCC_13 | d32 | MFCC_13__d32 |
| 250 | MFCC_13 | log_t1 | MFCC_13__log_t1 |
| 251 | MFCC_13 | log_t2 | MFCC_13__log_t2 |
| 252 | MFCC_13 | log_t3 | MFCC_13__log_t3 |
| 253 | MFCC_13 | logd21 | MFCC_13__logd21 |
| 254 | MFCC_13 | logd32 | MFCC_13__logd32 |
| 255 | MFCC_13 | mean3 | MFCC_13__mean3 |
| 256 | MFCC_13 | median3 | MFCC_13__median3 |
| 257 | MFCC_13 | pct21 | MFCC_13__pct21 |
| 258 | MFCC_13 | pct32 | MFCC_13__pct32 |
| 259 | MFCC_13 | r21 | MFCC_13__r21 |
| 260 | MFCC_13 | r32 | MFCC_13__r32 |
| 261 | MFCC_13 | range3 | MFCC_13__range3 |
| 262 | MFCC_13 | slope3 | MFCC_13__slope3 |
| 263 | MFCC_13 | std3 | MFCC_13__std3 |
| 264 | MFCC_13 | t1 | MFCC_13__t1 |
| 265 | MFCC_13 | t2 | MFCC_13__t2 |
| 266 | MFCC_13 | t3 | MFCC_13__t3 |
| 267 | MFCC_2 | d21 | MFCC_2__d21 |
| 268 | MFCC_2 | d32 | MFCC_2__d32 |
| 269 | MFCC_2 | log_t1 | MFCC_2__log_t1 |
| 270 | MFCC_2 | log_t2 | MFCC_2__log_t2 |
| 271 | MFCC_2 | log_t3 | MFCC_2__log_t3 |
| 272 | MFCC_2 | logd21 | MFCC_2__logd21 |
| 273 | MFCC_2 | logd32 | MFCC_2__logd32 |
| 274 | MFCC_2 | mean3 | MFCC_2__mean3 |
| 275 | MFCC_2 | median3 | MFCC_2__median3 |
| 276 | MFCC_2 | pct21 | MFCC_2__pct21 |
| 277 | MFCC_2 | pct32 | MFCC_2__pct32 |
| 278 | MFCC_2 | r21 | MFCC_2__r21 |
| 279 | MFCC_2 | r32 | MFCC_2__r32 |
| 280 | MFCC_2 | range3 | MFCC_2__range3 |
| 281 | MFCC_2 | slope3 | MFCC_2__slope3 |
| 282 | MFCC_2 | std3 | MFCC_2__std3 |
| 283 | MFCC_2 | t1 | MFCC_2__t1 |
| 284 | MFCC_2 | t2 | MFCC_2__t2 |
| 285 | MFCC_2 | t3 | MFCC_2__t3 |
| 286 | MFCC_3 | d21 | MFCC_3__d21 |
| 287 | MFCC_3 | d32 | MFCC_3__d32 |
| 288 | MFCC_3 | log_t1 | MFCC_3__log_t1 |
| 289 | MFCC_3 | log_t2 | MFCC_3__log_t2 |
| 290 | MFCC_3 | log_t3 | MFCC_3__log_t3 |
| 291 | MFCC_3 | logd21 | MFCC_3__logd21 |
| 292 | MFCC_3 | logd32 | MFCC_3__logd32 |
| 293 | MFCC_3 | mean3 | MFCC_3__mean3 |
| 294 | MFCC_3 | median3 | MFCC_3__median3 |
| 295 | MFCC_3 | pct21 | MFCC_3__pct21 |
| 296 | MFCC_3 | pct32 | MFCC_3__pct32 |
| 297 | MFCC_3 | r21 | MFCC_3__r21 |
| 298 | MFCC_3 | r32 | MFCC_3__r32 |
| 299 | MFCC_3 | range3 | MFCC_3__range3 |
| 300 | MFCC_3 | slope3 | MFCC_3__slope3 |
| 301 | MFCC_3 | std3 | MFCC_3__std3 |
| 302 | MFCC_3 | t1 | MFCC_3__t1 |
| 303 | MFCC_3 | t2 | MFCC_3__t2 |
| 304 | MFCC_3 | t3 | MFCC_3__t3 |
| 305 | MFCC_4 | d21 | MFCC_4__d21 |
| 306 | MFCC_4 | d32 | MFCC_4__d32 |
| 307 | MFCC_4 | log_t1 | MFCC_4__log_t1 |
| 308 | MFCC_4 | log_t2 | MFCC_4__log_t2 |
| 309 | MFCC_4 | log_t3 | MFCC_4__log_t3 |
| 310 | MFCC_4 | logd21 | MFCC_4__logd21 |
| 311 | MFCC_4 | logd32 | MFCC_4__logd32 |
| 312 | MFCC_4 | mean3 | MFCC_4__mean3 |
| 313 | MFCC_4 | median3 | MFCC_4__median3 |
| 314 | MFCC_4 | pct21 | MFCC_4__pct21 |
| 315 | MFCC_4 | pct32 | MFCC_4__pct32 |
| 316 | MFCC_4 | r21 | MFCC_4__r21 |
| 317 | MFCC_4 | r32 | MFCC_4__r32 |
| 318 | MFCC_4 | range3 | MFCC_4__range3 |
| 319 | MFCC_4 | slope3 | MFCC_4__slope3 |
| 320 | MFCC_4 | std3 | MFCC_4__std3 |
| 321 | MFCC_4 | t1 | MFCC_4__t1 |
| 322 | MFCC_4 | t2 | MFCC_4__t2 |
| 323 | MFCC_4 | t3 | MFCC_4__t3 |
| 324 | MFCC_5 | d21 | MFCC_5__d21 |
| 325 | MFCC_5 | d32 | MFCC_5__d32 |
| 326 | MFCC_5 | log_t1 | MFCC_5__log_t1 |
| 327 | MFCC_5 | log_t2 | MFCC_5__log_t2 |
| 328 | MFCC_5 | log_t3 | MFCC_5__log_t3 |
| 329 | MFCC_5 | logd21 | MFCC_5__logd21 |
| 330 | MFCC_5 | logd32 | MFCC_5__logd32 |
| 331 | MFCC_5 | mean3 | MFCC_5__mean3 |
| 332 | MFCC_5 | median3 | MFCC_5__median3 |
| 333 | MFCC_5 | pct21 | MFCC_5__pct21 |
| 334 | MFCC_5 | pct32 | MFCC_5__pct32 |
| 335 | MFCC_5 | r21 | MFCC_5__r21 |
| 336 | MFCC_5 | r32 | MFCC_5__r32 |
| 337 | MFCC_5 | range3 | MFCC_5__range3 |
| 338 | MFCC_5 | slope3 | MFCC_5__slope3 |
| 339 | MFCC_5 | std3 | MFCC_5__std3 |
| 340 | MFCC_5 | t1 | MFCC_5__t1 |
| 341 | MFCC_5 | t2 | MFCC_5__t2 |
| 342 | MFCC_5 | t3 | MFCC_5__t3 |
| 343 | MFCC_6 | d21 | MFCC_6__d21 |
| 344 | MFCC_6 | d32 | MFCC_6__d32 |
| 345 | MFCC_6 | log_t1 | MFCC_6__log_t1 |
| 346 | MFCC_6 | log_t2 | MFCC_6__log_t2 |
| 347 | MFCC_6 | log_t3 | MFCC_6__log_t3 |
| 348 | MFCC_6 | logd21 | MFCC_6__logd21 |
| 349 | MFCC_6 | logd32 | MFCC_6__logd32 |
| 350 | MFCC_6 | mean3 | MFCC_6__mean3 |
| 351 | MFCC_6 | median3 | MFCC_6__median3 |
| 352 | MFCC_6 | pct21 | MFCC_6__pct21 |
| 353 | MFCC_6 | pct32 | MFCC_6__pct32 |
| 354 | MFCC_6 | r21 | MFCC_6__r21 |
| 355 | MFCC_6 | r32 | MFCC_6__r32 |
| 356 | MFCC_6 | range3 | MFCC_6__range3 |
| 357 | MFCC_6 | slope3 | MFCC_6__slope3 |
| 358 | MFCC_6 | std3 | MFCC_6__std3 |
| 359 | MFCC_6 | t1 | MFCC_6__t1 |
| 360 | MFCC_6 | t2 | MFCC_6__t2 |
| 361 | MFCC_6 | t3 | MFCC_6__t3 |
| 362 | MFCC_7 | d21 | MFCC_7__d21 |
| 363 | MFCC_7 | d32 | MFCC_7__d32 |
| 364 | MFCC_7 | log_t1 | MFCC_7__log_t1 |
| 365 | MFCC_7 | log_t2 | MFCC_7__log_t2 |
| 366 | MFCC_7 | log_t3 | MFCC_7__log_t3 |
| 367 | MFCC_7 | logd21 | MFCC_7__logd21 |
| 368 | MFCC_7 | logd32 | MFCC_7__logd32 |
| 369 | MFCC_7 | mean3 | MFCC_7__mean3 |
| 370 | MFCC_7 | median3 | MFCC_7__median3 |
| 371 | MFCC_7 | pct21 | MFCC_7__pct21 |
| 372 | MFCC_7 | pct32 | MFCC_7__pct32 |
| 373 | MFCC_7 | r21 | MFCC_7__r21 |
| 374 | MFCC_7 | r32 | MFCC_7__r32 |
| 375 | MFCC_7 | range3 | MFCC_7__range3 |
| 376 | MFCC_7 | slope3 | MFCC_7__slope3 |
| 377 | MFCC_7 | std3 | MFCC_7__std3 |
| 378 | MFCC_7 | t1 | MFCC_7__t1 |
| 379 | MFCC_7 | t2 | MFCC_7__t2 |
| 380 | MFCC_7 | t3 | MFCC_7__t3 |
| 381 | MFCC_8 | d21 | MFCC_8__d21 |
| 382 | MFCC_8 | d32 | MFCC_8__d32 |
| 383 | MFCC_8 | log_t1 | MFCC_8__log_t1 |
| 384 | MFCC_8 | log_t2 | MFCC_8__log_t2 |
| 385 | MFCC_8 | log_t3 | MFCC_8__log_t3 |
| 386 | MFCC_8 | logd21 | MFCC_8__logd21 |
| 387 | MFCC_8 | logd32 | MFCC_8__logd32 |
| 388 | MFCC_8 | mean3 | MFCC_8__mean3 |
| 389 | MFCC_8 | median3 | MFCC_8__median3 |
| 390 | MFCC_8 | pct21 | MFCC_8__pct21 |
| 391 | MFCC_8 | pct32 | MFCC_8__pct32 |
| 392 | MFCC_8 | r21 | MFCC_8__r21 |
| 393 | MFCC_8 | r32 | MFCC_8__r32 |
| 394 | MFCC_8 | range3 | MFCC_8__range3 |
| 395 | MFCC_8 | slope3 | MFCC_8__slope3 |
| 396 | MFCC_8 | std3 | MFCC_8__std3 |
| 397 | MFCC_8 | t1 | MFCC_8__t1 |
| 398 | MFCC_8 | t2 | MFCC_8__t2 |
| 399 | MFCC_8 | t3 | MFCC_8__t3 |
| 400 | MFCC_9 | d21 | MFCC_9__d21 |
| 401 | MFCC_9 | d32 | MFCC_9__d32 |
| 402 | MFCC_9 | log_t1 | MFCC_9__log_t1 |
| 403 | MFCC_9 | log_t2 | MFCC_9__log_t2 |
| 404 | MFCC_9 | log_t3 | MFCC_9__log_t3 |
| 405 | MFCC_9 | logd21 | MFCC_9__logd21 |
| 406 | MFCC_9 | logd32 | MFCC_9__logd32 |
| 407 | MFCC_9 | mean3 | MFCC_9__mean3 |
| 408 | MFCC_9 | median3 | MFCC_9__median3 |
| 409 | MFCC_9 | pct21 | MFCC_9__pct21 |
| 410 | MFCC_9 | pct32 | MFCC_9__pct32 |
| 411 | MFCC_9 | r21 | MFCC_9__r21 |
| 412 | MFCC_9 | r32 | MFCC_9__r32 |
| 413 | MFCC_9 | range3 | MFCC_9__range3 |
| 414 | MFCC_9 | slope3 | MFCC_9__slope3 |
| 415 | MFCC_9 | std3 | MFCC_9__std3 |
| 416 | MFCC_9 | t1 | MFCC_9__t1 |
| 417 | MFCC_9 | t2 | MFCC_9__t2 |
| 418 | MFCC_9 | t3 | MFCC_9__t3 |
| 419 | Mean Amplitude | d21 | Mean Amplitude__d21 |
| 420 | Mean Amplitude | d32 | Mean Amplitude__d32 |
| 421 | Mean Amplitude | log_t1 | Mean Amplitude__log_t1 |
| 422 | Mean Amplitude | log_t2 | Mean Amplitude__log_t2 |
| 423 | Mean Amplitude | log_t3 | Mean Amplitude__log_t3 |
| 424 | Mean Amplitude | logd21 | Mean Amplitude__logd21 |
| 425 | Mean Amplitude | logd32 | Mean Amplitude__logd32 |
| 426 | Mean Amplitude | mean3 | Mean Amplitude__mean3 |
| 427 | Mean Amplitude | median3 | Mean Amplitude__median3 |
| 428 | Mean Amplitude | pct21 | Mean Amplitude__pct21 |
| 429 | Mean Amplitude | pct32 | Mean Amplitude__pct32 |
| 430 | Mean Amplitude | r21 | Mean Amplitude__r21 |
| 431 | Mean Amplitude | r32 | Mean Amplitude__r32 |
| 432 | Mean Amplitude | range3 | Mean Amplitude__range3 |
| 433 | Mean Amplitude | slope3 | Mean Amplitude__slope3 |
| 434 | Mean Amplitude | std3 | Mean Amplitude__std3 |
| 435 | Mean Amplitude | t1 | Mean Amplitude__t1 |
| 436 | Mean Amplitude | t2 | Mean Amplitude__t2 |
| 437 | Mean Amplitude | t3 | Mean Amplitude__t3 |
| 438 | Mean RMS | d21 | Mean RMS__d21 |
| 439 | Mean RMS | d32 | Mean RMS__d32 |
| 440 | Mean RMS | log_t1 | Mean RMS__log_t1 |
| 441 | Mean RMS | log_t2 | Mean RMS__log_t2 |
| 442 | Mean RMS | log_t3 | Mean RMS__log_t3 |
| 443 | Mean RMS | logd21 | Mean RMS__logd21 |
| 444 | Mean RMS | logd32 | Mean RMS__logd32 |
| 445 | Mean RMS | mean3 | Mean RMS__mean3 |
| 446 | Mean RMS | median3 | Mean RMS__median3 |
| 447 | Mean RMS | pct21 | Mean RMS__pct21 |
| 448 | Mean RMS | pct32 | Mean RMS__pct32 |
| 449 | Mean RMS | r21 | Mean RMS__r21 |
| 450 | Mean RMS | r32 | Mean RMS__r32 |
| 451 | Mean RMS | range3 | Mean RMS__range3 |
| 452 | Mean RMS | slope3 | Mean RMS__slope3 |
| 453 | Mean RMS | std3 | Mean RMS__std3 |
| 454 | Mean RMS | t1 | Mean RMS__t1 |
| 455 | Mean RMS | t2 | Mean RMS__t2 |
| 456 | Mean RMS | t3 | Mean RMS__t3 |
| 457 | Mean Spectral Bandwidth (Hz) | d21 | Mean Spectral Bandwidth (Hz)__d21 |
| 458 | Mean Spectral Bandwidth (Hz) | d32 | Mean Spectral Bandwidth (Hz)__d32 |
| 459 | Mean Spectral Bandwidth (Hz) | log_t1 | Mean Spectral Bandwidth (Hz)__log_t1 |
| 460 | Mean Spectral Bandwidth (Hz) | log_t2 | Mean Spectral Bandwidth (Hz)__log_t2 |
| 461 | Mean Spectral Bandwidth (Hz) | log_t3 | Mean Spectral Bandwidth (Hz)__log_t3 |
| 462 | Mean Spectral Bandwidth (Hz) | logd21 | Mean Spectral Bandwidth (Hz)__logd21 |
| 463 | Mean Spectral Bandwidth (Hz) | logd32 | Mean Spectral Bandwidth (Hz)__logd32 |
| 464 | Mean Spectral Bandwidth (Hz) | mean3 | Mean Spectral Bandwidth (Hz)__mean3 |
| 465 | Mean Spectral Bandwidth (Hz) | median3 | Mean Spectral Bandwidth (Hz)__median3 |
| 466 | Mean Spectral Bandwidth (Hz) | pct21 | Mean Spectral Bandwidth (Hz)__pct21 |
| 467 | Mean Spectral Bandwidth (Hz) | pct32 | Mean Spectral Bandwidth (Hz)__pct32 |
| 468 | Mean Spectral Bandwidth (Hz) | r21 | Mean Spectral Bandwidth (Hz)__r21 |
| 469 | Mean Spectral Bandwidth (Hz) | r32 | Mean Spectral Bandwidth (Hz)__r32 |
| 470 | Mean Spectral Bandwidth (Hz) | range3 | Mean Spectral Bandwidth (Hz)__range3 |
| 471 | Mean Spectral Bandwidth (Hz) | slope3 | Mean Spectral Bandwidth (Hz)__slope3 |
| 472 | Mean Spectral Bandwidth (Hz) | std3 | Mean Spectral Bandwidth (Hz)__std3 |
| 473 | Mean Spectral Bandwidth (Hz) | t1 | Mean Spectral Bandwidth (Hz)__t1 |
| 474 | Mean Spectral Bandwidth (Hz) | t2 | Mean Spectral Bandwidth (Hz)__t2 |
| 475 | Mean Spectral Bandwidth (Hz) | t3 | Mean Spectral Bandwidth (Hz)__t3 |
| 476 | Mean Spectral Centroid (Hz) | d21 | Mean Spectral Centroid (Hz)__d21 |
| 477 | Mean Spectral Centroid (Hz) | d32 | Mean Spectral Centroid (Hz)__d32 |
| 478 | Mean Spectral Centroid (Hz) | log_t1 | Mean Spectral Centroid (Hz)__log_t1 |
| 479 | Mean Spectral Centroid (Hz) | log_t2 | Mean Spectral Centroid (Hz)__log_t2 |
| 480 | Mean Spectral Centroid (Hz) | log_t3 | Mean Spectral Centroid (Hz)__log_t3 |
| 481 | Mean Spectral Centroid (Hz) | logd21 | Mean Spectral Centroid (Hz)__logd21 |
| 482 | Mean Spectral Centroid (Hz) | logd32 | Mean Spectral Centroid (Hz)__logd32 |
| 483 | Mean Spectral Centroid (Hz) | mean3 | Mean Spectral Centroid (Hz)__mean3 |
| 484 | Mean Spectral Centroid (Hz) | median3 | Mean Spectral Centroid (Hz)__median3 |
| 485 | Mean Spectral Centroid (Hz) | pct21 | Mean Spectral Centroid (Hz)__pct21 |
| 486 | Mean Spectral Centroid (Hz) | pct32 | Mean Spectral Centroid (Hz)__pct32 |
| 487 | Mean Spectral Centroid (Hz) | r21 | Mean Spectral Centroid (Hz)__r21 |
| 488 | Mean Spectral Centroid (Hz) | r32 | Mean Spectral Centroid (Hz)__r32 |
| 489 | Mean Spectral Centroid (Hz) | range3 | Mean Spectral Centroid (Hz)__range3 |
| 490 | Mean Spectral Centroid (Hz) | slope3 | Mean Spectral Centroid (Hz)__slope3 |
| 491 | Mean Spectral Centroid (Hz) | std3 | Mean Spectral Centroid (Hz)__std3 |
| 492 | Mean Spectral Centroid (Hz) | t1 | Mean Spectral Centroid (Hz)__t1 |
| 493 | Mean Spectral Centroid (Hz) | t2 | Mean Spectral Centroid (Hz)__t2 |
| 494 | Mean Spectral Centroid (Hz) | t3 | Mean Spectral Centroid (Hz)__t3 |
| 495 | Mean Spectral Flatness | d21 | Mean Spectral Flatness__d21 |
| 496 | Mean Spectral Flatness | d32 | Mean Spectral Flatness__d32 |
| 497 | Mean Spectral Flatness | log_t1 | Mean Spectral Flatness__log_t1 |
| 498 | Mean Spectral Flatness | log_t2 | Mean Spectral Flatness__log_t2 |
| 499 | Mean Spectral Flatness | log_t3 | Mean Spectral Flatness__log_t3 |
| 500 | Mean Spectral Flatness | logd21 | Mean Spectral Flatness__logd21 |
| 501 | Mean Spectral Flatness | logd32 | Mean Spectral Flatness__logd32 |
| 502 | Mean Spectral Flatness | mean3 | Mean Spectral Flatness__mean3 |
| 503 | Mean Spectral Flatness | median3 | Mean Spectral Flatness__median3 |
| 504 | Mean Spectral Flatness | pct21 | Mean Spectral Flatness__pct21 |
| 505 | Mean Spectral Flatness | pct32 | Mean Spectral Flatness__pct32 |
| 506 | Mean Spectral Flatness | r21 | Mean Spectral Flatness__r21 |
| 507 | Mean Spectral Flatness | r32 | Mean Spectral Flatness__r32 |
| 508 | Mean Spectral Flatness | range3 | Mean Spectral Flatness__range3 |
| 509 | Mean Spectral Flatness | slope3 | Mean Spectral Flatness__slope3 |
| 510 | Mean Spectral Flatness | std3 | Mean Spectral Flatness__std3 |
| 511 | Mean Spectral Flatness | t1 | Mean Spectral Flatness__t1 |
| 512 | Mean Spectral Flatness | t2 | Mean Spectral Flatness__t2 |
| 513 | Mean Spectral Flatness | t3 | Mean Spectral Flatness__t3 |
| 514 | Mean Spectral Roll-off (Hz, 85%) | d21 | Mean Spectral Roll-off (Hz, 85%)__d21 |
| 515 | Mean Spectral Roll-off (Hz, 85%) | d32 | Mean Spectral Roll-off (Hz, 85%)__d32 |
| 516 | Mean Spectral Roll-off (Hz, 85%) | log_t1 | Mean Spectral Roll-off (Hz, 85%)__log_t1 |
| 517 | Mean Spectral Roll-off (Hz, 85%) | log_t2 | Mean Spectral Roll-off (Hz, 85%)__log_t2 |
| 518 | Mean Spectral Roll-off (Hz, 85%) | log_t3 | Mean Spectral Roll-off (Hz, 85%)__log_t3 |
| 519 | Mean Spectral Roll-off (Hz, 85%) | logd21 | Mean Spectral Roll-off (Hz, 85%)__logd21 |
| 520 | Mean Spectral Roll-off (Hz, 85%) | logd32 | Mean Spectral Roll-off (Hz, 85%)__logd32 |
| 521 | Mean Spectral Roll-off (Hz, 85%) | mean3 | Mean Spectral Roll-off (Hz, 85%)__mean3 |
| 522 | Mean Spectral Roll-off (Hz, 85%) | median3 | Mean Spectral Roll-off (Hz, 85%)__median3 |
| 523 | Mean Spectral Roll-off (Hz, 85%) | pct21 | Mean Spectral Roll-off (Hz, 85%)__pct21 |
| 524 | Mean Spectral Roll-off (Hz, 85%) | pct32 | Mean Spectral Roll-off (Hz, 85%)__pct32 |
| 525 | Mean Spectral Roll-off (Hz, 85%) | r21 | Mean Spectral Roll-off (Hz, 85%)__r21 |
| 526 | Mean Spectral Roll-off (Hz, 85%) | r32 | Mean Spectral Roll-off (Hz, 85%)__r32 |
| 527 | Mean Spectral Roll-off (Hz, 85%) | range3 | Mean Spectral Roll-off (Hz, 85%)__range3 |
| 528 | Mean Spectral Roll-off (Hz, 85%) | slope3 | Mean Spectral Roll-off (Hz, 85%)__slope3 |
| 529 | Mean Spectral Roll-off (Hz, 85%) | std3 | Mean Spectral Roll-off (Hz, 85%)__std3 |
| 530 | Mean Spectral Roll-off (Hz, 85%) | t1 | Mean Spectral Roll-off (Hz, 85%)__t1 |
| 531 | Mean Spectral Roll-off (Hz, 85%) | t2 | Mean Spectral Roll-off (Hz, 85%)__t2 |
| 532 | Mean Spectral Roll-off (Hz, 85%) | t3 | Mean Spectral Roll-off (Hz, 85%)__t3 |
| 533 | Mean Zero Crossing Rate | d21 | Mean Zero Crossing Rate__d21 |
| 534 | Mean Zero Crossing Rate | d32 | Mean Zero Crossing Rate__d32 |
| 535 | Mean Zero Crossing Rate | log_t1 | Mean Zero Crossing Rate__log_t1 |
| 536 | Mean Zero Crossing Rate | log_t2 | Mean Zero Crossing Rate__log_t2 |
| 537 | Mean Zero Crossing Rate | log_t3 | Mean Zero Crossing Rate__log_t3 |
| 538 | Mean Zero Crossing Rate | logd21 | Mean Zero Crossing Rate__logd21 |
| 539 | Mean Zero Crossing Rate | logd32 | Mean Zero Crossing Rate__logd32 |
| 540 | Mean Zero Crossing Rate | mean3 | Mean Zero Crossing Rate__mean3 |
| 541 | Mean Zero Crossing Rate | median3 | Mean Zero Crossing Rate__median3 |
| 542 | Mean Zero Crossing Rate | pct21 | Mean Zero Crossing Rate__pct21 |
| 543 | Mean Zero Crossing Rate | pct32 | Mean Zero Crossing Rate__pct32 |
| 544 | Mean Zero Crossing Rate | r21 | Mean Zero Crossing Rate__r21 |
| 545 | Mean Zero Crossing Rate | r32 | Mean Zero Crossing Rate__r32 |
| 546 | Mean Zero Crossing Rate | range3 | Mean Zero Crossing Rate__range3 |
| 547 | Mean Zero Crossing Rate | slope3 | Mean Zero Crossing Rate__slope3 |
| 548 | Mean Zero Crossing Rate | std3 | Mean Zero Crossing Rate__std3 |
| 549 | Mean Zero Crossing Rate | t1 | Mean Zero Crossing Rate__t1 |
| 550 | Mean Zero Crossing Rate | t2 | Mean Zero Crossing Rate__t2 |
| 551 | Mean Zero Crossing Rate | t3 | Mean Zero Crossing Rate__t3 |
| 552 | Peaks Detected | d21 | Peaks Detected__d21 |
| 553 | Peaks Detected | d32 | Peaks Detected__d32 |
| 554 | Peaks Detected | log_t1 | Peaks Detected__log_t1 |
| 555 | Peaks Detected | log_t2 | Peaks Detected__log_t2 |
| 556 | Peaks Detected | log_t3 | Peaks Detected__log_t3 |
| 557 | Peaks Detected | logd21 | Peaks Detected__logd21 |
| 558 | Peaks Detected | logd32 | Peaks Detected__logd32 |
| 559 | Peaks Detected | mean3 | Peaks Detected__mean3 |
| 560 | Peaks Detected | median3 | Peaks Detected__median3 |
| 561 | Peaks Detected | pct21 | Peaks Detected__pct21 |
| 562 | Peaks Detected | pct32 | Peaks Detected__pct32 |
| 563 | Peaks Detected | r21 | Peaks Detected__r21 |
| 564 | Peaks Detected | r32 | Peaks Detected__r32 |
| 565 | Peaks Detected | range3 | Peaks Detected__range3 |
| 566 | Peaks Detected | slope3 | Peaks Detected__slope3 |
| 567 | Peaks Detected | std3 | Peaks Detected__std3 |
| 568 | Peaks Detected | t1 | Peaks Detected__t1 |
| 569 | Peaks Detected | t2 | Peaks Detected__t2 |
| 570 | Peaks Detected | t3 | Peaks Detected__t3 |
