# Supplementary material for: Acoustic cues into a surgeon-assist physical AI for detecting bone penetration during spinal surgery
Source: Sci Rep. 2026 Apr 19;16:18113. doi: 10.1038/s41598-026-48857-w (PMC13254284; doi:10.1038/s41598-026-48857-w)
Supplement: Supplementary file 3 — Supplementary Material 3 [file 41598_2026_48857_MOESM3_ESM.docx]

| **Supplementary Table 3. Feature importance of the model.** | | |
| --- | --- | --- |
| **Ranking** | **Feature** | **Importance** |
| 1 | MFCC_3__slope3 | 1294 |
| 2 | MFCC_2__slope3 | 501 |
| 3 | MFCC_2__range3 | 310 |
| 4 | Contrast_7 (dB)__t3 | 280 |
| 5 | MFCC_3__r21 | 261 |
| 6 | Mean Zero Crossing Rate__std3 | 176 |
| 7 | MFCC_10__range3 | 146 |
| 8 | MFCC_5__t1 | 144 |
| 9 | Contrast_1 (dB)__median3 | 136 |
| 10 | MFCC_3__d21 | 134 |
| 11 | Mean Spectral Bandwidth (Hz)__slope3 | 133 |
| 12 | MFCC_5__median3 | 132 |
| 13 | Contrast_2 (dB)__std3 | 123 |
| 14 | Mean Amplitude__t1 | 110 |
| 15 | MFCC_13__d21 | 97 |
| 16 | MFCC_5__range3 | 94 |
| 17 | Mean Zero Crossing Rate__slope3 | 90 |
| 18 | MFCC_2__d21 | 88 |
| 19 | MFCC_13__std3 | 87 |
| 20 | MFCC_13__t3 | 85 |
| 21 | Contrast_7 (dB)__median3 | 82 |
| 22 | Mean Spectral Centroid (Hz)__slope3 | 81 |
| 23 | Mean RMS__slope3 | 78 |
| 24 | MFCC_5__std3 | 77 |
| 25 | Mean Amplitude__slope3 | 76 |
| 26 | MFCC_13__t2 | 74 |
| 27 | Contrast_5 (dB)__t3 | 73 |
| 28 | MFCC_1__slope3 | 70 |
| 29 | MFCC_4__range3 | 69 |
| 30 | MFCC_5__t3 | 68 |
| 31 | MFCC_6__median3 | 67 |
| 32 | MFCC_13__r21 | 65 |
| 33 | Contrast_2 (dB)__range3 | 64 |
| 34 | MFCC_2__std3 | 60 |
| 35 | MFCC_13__slope3 | 59 |
| 36 | Contrast_4 (dB)__d21 | 59 |
| 37 | Mean Amplitude__t3 | 59 |
| 38 | MFCC_3__std3 | 57 |
| 39 | MFCC_12__t2 | 56 |
| 40 | Mean Zero Crossing Rate__t1 | 56 |
| 41 | MFCC_12__d32 | 56 |
| 42 | Mean Zero Crossing Rate__d32 | 56 |
| 43 | Mean Amplitude__range3 | 56 |
| 44 | Duration (s)__t3 | 54 |
| 45 | MFCC_4__d21 | 53 |
| 46 | MFCC_4__slope3 | 53 |
| 47 | MFCC_13__range3 | 53 |
| 48 | Contrast_1 (dB)__slope3 | 52 |
| 49 | Contrast_5 (dB)__median3 | 52 |
| 50 | Contrast_3 (dB)__std3 | 52 |
| 51 | MFCC_12__range3 | 51 |
| 52 | MFCC_11__r21 | 51 |
| 53 | Contrast_3 (dB)__median3 | 51 |
| 54 | Mean Spectral Bandwidth (Hz)__d21 | 50 |
| 55 | MFCC_1__t2 | 49 |
| 56 | MFCC_6__std3 | 49 |
| 57 | MFCC_5__r21 | 48 |
| 58 | Mean Zero Crossing Rate__mean3 | 48 |
| 59 | MFCC_10__slope3 | 47 |
| 60 | MFCC_3__d32 | 47 |
| 61 | Mean Spectral Roll-off (Hz, 85%)__t1 | 45 |
| 62 | MFCC_12__logd21 | 45 |
| 63 | MFCC_11__std3 | 44 |
| 64 | MFCC_1__pct32 | 43 |
| 65 | Mean Zero Crossing Rate__range3 | 43 |
| 66 | Mean Spectral Roll-off (Hz, 85%)__r21 | 42 |
| 67 | MFCC_12__d21 | 41 |
| 68 | Mean Spectral Flatness__d21 | 41 |
| 69 | MFCC_8__pct21 | 41 |
| 70 | Contrast_3 (dB)__slope3 | 40 |
| 71 | MFCC_7__slope3 | 40 |
| 72 | Contrast_1 (dB)__t3 | 40 |
| 73 | MFCC_3__range3 | 39 |
| 74 | MFCC_7__d21 | 39 |
| 75 | MFCC_5__d21 | 38 |
| 76 | Mean Spectral Flatness__pct21 | 38 |
| 77 | Contrast_4 (dB)__r32 | 37 |
| 78 | MFCC_2__r21 | 37 |
| 79 | MFCC_12__t3 | 37 |
| 80 | Contrast_5 (dB)__t1 | 37 |
| 81 | Contrast_3 (dB)__mean3 | 37 |
| 82 | Contrast_7 (dB)__range3 | 37 |
| 83 | MFCC_5__pct21 | 36 |
| 84 | Contrast_6 (dB)__mean3 | 36 |
| 85 | Mean Spectral Centroid (Hz)__r21 | 36 |
| 86 | Duration (s)__r21 | 36 |
| 87 | MFCC_9__slope3 | 35 |
| 88 | Mean Spectral Centroid (Hz)__r32 | 35 |
| 89 | Contrast_4 (dB)__slope3 | 35 |
| 90 | MFCC_12__pct21 | 35 |
| 91 | Mean Spectral Bandwidth (Hz)__median3 | 35 |
| 92 | Energy__slope3 | 34 |
| 93 | Mean Spectral Centroid (Hz)__d32 | 34 |
| 94 | Duration (s)__t1 | 34 |
| 95 | MFCC_10__d21 | 34 |
| 96 | MFCC_10__std3 | 33 |
| 97 | Mean Spectral Flatness__range3 | 33 |
| 98 | MFCC_6__d32 | 33 |
| 99 | Contrast_3 (dB)__r21 | 33 |
| 100 | MFCC_2__d32 | 33 |
| 101 | MFCC_12__median3 | 32 |
| 102 | MFCC_12__slope3 | 32 |
| 103 | Contrast_7 (dB)__std3 | 32 |
| 104 | MFCC_5__slope3 | 31 |
| 105 | Contrast_5 (dB)__mean3 | 31 |
| 106 | Mean Spectral Roll-off (Hz, 85%)__std3 | 30 |
| 107 | MFCC_8__slope3 | 30 |
| 108 | MFCC_9__std3 | 30 |
| 109 | Contrast_1 (dB)__std3 | 30 |
| 110 | Mean Zero Crossing Rate__t3 | 30 |
| 111 | Contrast_7 (dB)__r32 | 29 |
| 112 | MFCC_7__r32 | 29 |
| 113 | MFCC_8__r21 | 29 |
| 114 | Energy__t3 | 29 |
| 115 | MFCC_8__std3 | 29 |
| 116 | MFCC_3__t1 | 29 |
| 117 | Contrast_4 (dB)__r21 | 28 |
| 118 | MFCC_9__median3 | 28 |
| 119 | Mean Zero Crossing Rate__logd32 | 28 |
| 120 | Contrast_4 (dB)__logd21 | 28 |
| 121 | MFCC_13__r32 | 27 |
| 122 | Duration (s)__d32 | 27 |
| 123 | MFCC_2__logd21 | 27 |
| 124 | MFCC_11__d32 | 27 |
| 125 | Contrast_4 (dB)__std3 | 26 |
| 126 | MFCC_11__median3 | 26 |
| 127 | MFCC_12__r32 | 26 |
| 128 | Contrast_1 (dB)__d32 | 26 |
| 129 | MFCC_12__std3 | 26 |
| 130 | MFCC_8__range3 | 25 |
| 131 | Contrast_4 (dB)__d32 | 25 |
| 132 | Contrast_3 (dB)__d21 | 25 |
| 133 | Contrast_6 (dB)__std3 | 25 |
| 134 | Mean Spectral Roll-off (Hz, 85%)__d21 | 25 |
| 135 | Mean Amplitude__log_t2 | 24 |
| 136 | Contrast_3 (dB)__d32 | 24 |
| 137 | Mean Spectral Bandwidth (Hz)__d32 | 24 |
| 138 | Contrast_2 (dB)__t1 | 24 |
| 139 | MFCC_9__logd32 | 24 |
| 140 | MFCC_10__t1 | 23 |
| 141 | Mean Spectral Roll-off (Hz, 85%)__range3 | 23 |
| 142 | Contrast_6 (dB)__range3 | 23 |
| 143 | Mean Amplitude__std3 | 23 |
| 144 | MFCC_5__t2 | 22 |
| 145 | MFCC_6__t3 | 22 |
| 146 | Mean Amplitude__logd21 | 22 |
| 147 | Mean RMS__d32 | 22 |
| 148 | Mean Spectral Flatness__t1 | 22 |
| 149 | MFCC_6__r32 | 21 |
| 150 | MFCC_9__mean3 | 21 |
| 151 | Contrast_2 (dB)__r21 | 21 |
| 152 | Contrast_1 (dB)__mean3 | 21 |
| 153 | MFCC_7__t2 | 21 |
| 154 | MFCC_5__logd21 | 21 |
| 155 | Contrast_6 (dB)__r32 | 21 |
| 156 | Energy__r21 | 21 |
| 157 | Mean Spectral Centroid (Hz)__t1 | 20 |
| 158 | MFCC_2__log_t3 | 20 |
| 159 | MFCC_1__r32 | 20 |
| 160 | Mean Zero Crossing Rate__median3 | 20 |
| 161 | Mean Zero Crossing Rate__r32 | 20 |
| 162 | MFCC_4__d32 | 20 |
| 163 | MFCC_9__log_t1 | 20 |
| 164 | Contrast_5 (dB)__t2 | 20 |
| 165 | Contrast_6 (dB)__t2 | 19 |
| 166 | MFCC_2__pct21 | 19 |
| 167 | MFCC_6__range3 | 19 |
| 168 | MFCC_5__mean3 | 19 |
| 169 | Contrast_6 (dB)__t1 | 19 |
| 170 | Contrast_3 (dB)__t2 | 19 |
| 171 | MFCC_11__t2 | 18 |
| 172 | Contrast_6 (dB)__slope3 | 18 |
| 173 | MFCC_9__t2 | 18 |
| 174 | Mean Spectral Bandwidth (Hz)__t1 | 18 |
| 175 | MFCC_12__t1 | 18 |
| 176 | Contrast_7 (dB)__t1 | 18 |
| 177 | MFCC_9__r21 | 17 |
| 178 | Mean Amplitude__log_t1 | 17 |
| 179 | Contrast_1 (dB)__t1 | 17 |
| 180 | MFCC_6__d21 | 17 |
| 181 | MFCC_9__r32 | 17 |
| 182 | Mean Spectral Flatness__std3 | 17 |
| 183 | Mean Spectral Roll-off (Hz, 85%)__slope3 | 17 |
| 184 | Energy__d32 | 17 |
| 185 | Mean Zero Crossing Rate__t2 | 17 |
| 186 | MFCC_6__pct21 | 16 |
| 187 | MFCC_2__r32 | 16 |
| 188 | MFCC_1__std3 | 16 |
| 189 | Duration (s)__range3 | 16 |
| 190 | Contrast_7 (dB)__r21 | 16 |
| 191 | Mean RMS__d21 | 16 |
| 192 | MFCC_3__t2 | 16 |
| 193 | MFCC_7__r21 | 16 |
| 194 | MFCC_9__d32 | 16 |
| 195 | Contrast_4 (dB)__t2 | 15 |
| 196 | Mean Spectral Flatness__median3 | 15 |
| 197 | Duration (s)__logd21 | 15 |
| 198 | MFCC_4__median3 | 15 |
| 199 | Mean RMS__mean3 | 15 |
| 200 | Contrast_4 (dB)__median3 | 15 |
| 201 | MFCC_7__t1 | 14 |
| 202 | MFCC_12__log_t1 | 14 |
| 203 | Contrast_6 (dB)__median3 | 14 |
| 204 | Mean Spectral Bandwidth (Hz)__std3 | 14 |
| 205 | MFCC_4__t3 | 14 |
| 206 | MFCC_2__t3 | 14 |
| 207 | MFCC_9__t3 | 14 |
| 208 | MFCC_10__r32 | 14 |
| 209 | Energy__t1 | 14 |
| 210 | MFCC_3__pct21 | 13 |
| 211 | Duration (s)__pct21 | 13 |
| 212 | Contrast_3 (dB)__pct32 | 13 |
| 213 | Mean Spectral Bandwidth (Hz)__r21 | 13 |
| 214 | Mean Spectral Centroid (Hz)__range3 | 13 |
| 215 | Mean Spectral Flatness__pct32 | 13 |
| 216 | MFCC_2__t1 | 13 |
| 217 | Contrast_6 (dB)__t3 | 13 |
| 218 | Duration (s)__std3 | 13 |
| 219 | Contrast_2 (dB)__t3 | 13 |
| 220 | Contrast_2 (dB)__d32 | 13 |
| 221 | Mean Spectral Roll-off (Hz, 85%)__d32 | 13 |
| 222 | Contrast_5 (dB)__r21 | 12 |
| 223 | MFCC_5__pct32 | 12 |
| 224 | MFCC_13__mean3 | 12 |
| 225 | MFCC_5__d32 | 12 |
| 226 | Contrast_6 (dB)__d32 | 12 |
| 227 | Duration (s)__t2 | 12 |
| 228 | MFCC_3__logd32 | 12 |
| 229 | Mean RMS__logd32 | 12 |
| 230 | Contrast_7 (dB)__log_t1 | 12 |
| 231 | Mean Spectral Bandwidth (Hz)__range3 | 12 |
| 232 | Contrast_2 (dB)__mean3 | 12 |
| 233 | Contrast_3 (dB)__t3 | 11 |
| 234 | Duration (s)__d21 | 11 |
| 235 | Contrast_6 (dB)__r21 | 11 |
| 236 | Contrast_5 (dB)__d32 | 11 |
| 237 | Contrast_7 (dB)__mean3 | 11 |
| 238 | Mean Spectral Centroid (Hz)__std3 | 11 |
| 239 | Mean Amplitude__t2 | 11 |
| 240 | Mean Amplitude__d32 | 11 |
| 241 | MFCC_10__t3 | 11 |
| 242 | Contrast_2 (dB)__median3 | 11 |
| 243 | Mean Amplitude__logd32 | 11 |
| 244 | MFCC_11__pct21 | 11 |
| 245 | Energy__t2 | 11 |
| 246 | Duration (s)__slope3 | 11 |
| 247 | Mean Spectral Flatness__slope3 | 11 |
| 248 | Contrast_5 (dB)__r32 | 11 |
| 249 | MFCC_10__pct32 | 10 |
| 250 | MFCC_11__t3 | 10 |
| 251 | MFCC_13__pct32 | 10 |
| 252 | MFCC_10__mean3 | 10 |
| 253 | Contrast_1 (dB)__r32 | 10 |
| 254 | Mean Amplitude__r21 | 10 |
| 255 | Contrast_1 (dB)__logd21 | 10 |
| 256 | Mean Spectral Bandwidth (Hz)__r32 | 10 |
| 257 | MFCC_9__pct32 | 10 |
| 258 | MFCC_9__range3 | 10 |
| 259 | Contrast_4 (dB)__t1 | 10 |
| 260 | Mean Zero Crossing Rate__d21 | 10 |
| 261 | MFCC_1__d32 | 10 |
| 262 | MFCC_1__t3 | 10 |
| 263 | Contrast_3 (dB)__logd21 | 10 |
| 264 | MFCC_11__r32 | 10 |
| 265 | MFCC_2__mean3 | 9 |
| 266 | MFCC_1__d21 | 9 |
| 267 | MFCC_7__logd21 | 9 |
| 268 | MFCC_9__t1 | 9 |
| 269 | Contrast_5 (dB)__d21 | 9 |
| 270 | MFCC_1__range3 | 9 |
| 271 | Energy__mean3 | 9 |
| 272 | MFCC_13__median3 | 9 |
| 273 | Contrast_7 (dB)__d21 | 9 |
| 274 | MFCC_7__pct32 | 9 |
| 275 | Energy__range3 | 9 |
| 276 | Contrast_1 (dB)__logd32 | 9 |
| 277 | Mean Spectral Centroid (Hz)__d21 | 8 |
| 278 | Contrast_5 (dB)__range3 | 8 |
| 279 | Contrast_3 (dB)__r32 | 8 |
| 280 | Mean Spectral Roll-off (Hz, 85%)__t3 | 8 |
| 281 | MFCC_7__log_t1 | 8 |
| 282 | Mean RMS__range3 | 8 |
| 283 | Contrast_5 (dB)__logd32 | 8 |
| 284 | MFCC_6__logd21 | 8 |
| 285 | Contrast_3 (dB)__range3 | 7 |
| 286 | Mean Spectral Flatness__r32 | 7 |
| 287 | Mean RMS__r21 | 7 |
| 288 | Contrast_4 (dB)__t3 | 7 |
| 289 | MFCC_4__std3 | 7 |
| 290 | MFCC_8__log_t2 | 7 |
| 291 | MFCC_8__d32 | 7 |
| 292 | Contrast_3 (dB)__t1 | 7 |
| 293 | Contrast_4 (dB)__mean3 | 7 |
| 294 | MFCC_10__median3 | 7 |
| 295 | MFCC_11__logd32 | 7 |
| 296 | Contrast_7 (dB)__d32 | 7 |
| 297 | MFCC_4__pct32 | 7 |
| 298 | MFCC_3__r32 | 7 |
| 299 | MFCC_8__d21 | 7 |
| 300 | MFCC_5__log_t1 | 7 |
| 301 | MFCC_11__slope3 | 6 |
| 302 | MFCC_6__slope3 | 6 |
| 303 | Mean Zero Crossing Rate__r21 | 6 |
| 304 | Mean RMS__r32 | 6 |
| 305 | MFCC_3__t3 | 6 |
| 306 | MFCC_6__r21 | 6 |
| 307 | MFCC_3__logd21 | 6 |
| 308 | Contrast_5 (dB)__log_t3 | 6 |
| 309 | MFCC_1__t1 | 6 |
| 310 | Mean Spectral Roll-off (Hz, 85%)__logd32 | 6 |
| 311 | MFCC_3__median3 | 6 |
| 312 | Mean RMS__t2 | 6 |
| 313 | Mean Zero Crossing Rate__pct21 | 6 |
| 314 | Contrast_3 (dB)__log_t2 | 6 |
| 315 | MFCC_4__pct21 | 6 |
| 316 | MFCC_8__logd21 | 5 |
| 317 | MFCC_10__d32 | 5 |
| 318 | MFCC_7__std3 | 5 |
| 319 | Contrast_1 (dB)__r21 | 5 |
| 320 | Mean Spectral Flatness__logd21 | 5 |
| 321 | MFCC_13__log_t3 | 5 |
| 322 | MFCC_8__median3 | 5 |
| 323 | Contrast_6 (dB)__d21 | 5 |
| 324 | Contrast_7 (dB)__t2 | 5 |
| 325 | Contrast_1 (dB)__d21 | 5 |
| 326 | Contrast_5 (dB)__slope3 | 5 |
| 327 | MFCC_10__pct21 | 5 |
| 328 | MFCC_1__logd32 | 5 |
| 329 | Contrast_2 (dB)__r32 | 5 |
| 330 | MFCC_11__pct32 | 5 |
| 331 | MFCC_11__logd21 | 4 |
| 332 | Mean Spectral Centroid (Hz)__t3 | 4 |
| 333 | Mean Spectral Flatness__t3 | 4 |
| 334 | MFCC_13__pct21 | 4 |
| 335 | MFCC_12__mean3 | 4 |
| 336 | MFCC_5__log_t2 | 4 |
| 337 | Energy__log_t1 | 4 |
| 338 | MFCC_4__logd21 | 4 |
| 339 | Contrast_7 (dB)__slope3 | 4 |
| 340 | Mean Spectral Roll-off (Hz, 85%)__t2 | 4 |
| 341 | MFCC_10__logd21 | 4 |
| 342 | Mean Spectral Roll-off (Hz, 85%)__mean3 | 4 |
| 343 | Contrast_2 (dB)__pct32 | 4 |
| 344 | Mean Zero Crossing Rate__log_t2 | 4 |
| 345 | Energy__pct21 | 4 |
| 346 | MFCC_12__logd32 | 4 |
| 347 | Contrast_4 (dB)__pct32 | 3 |
| 348 | Mean Spectral Bandwidth (Hz)__log_t1 | 3 |
| 349 | MFCC_10__r21 | 3 |
| 350 | Mean Spectral Bandwidth (Hz)__t2 | 3 |
| 351 | MFCC_4__r32 | 3 |
| 352 | Contrast_5 (dB)__pct32 | 3 |
| 353 | MFCC_8__r32 | 3 |
| 354 | MFCC_8__t2 | 3 |
| 355 | MFCC_2__pct32 | 3 |
| 356 | MFCC_7__logd32 | 3 |
| 357 | MFCC_11__log_t2 | 3 |
| 358 | Contrast_2 (dB)__d21 | 3 |
| 359 | MFCC_4__mean3 | 3 |
| 360 | MFCC_8__mean3 | 3 |
| 361 | Contrast_4 (dB)__range3 | 3 |
| 362 | MFCC_7__range3 | 3 |
| 363 | MFCC_12__r21 | 3 |
| 364 | Contrast_2 (dB)__logd32 | 2 |
| 365 | MFCC_11__range3 | 2 |
| 366 | MFCC_5__r32 | 2 |
| 367 | Mean Zero Crossing Rate__log_t3 | 2 |
| 368 | Mean Spectral Flatness__d32 | 2 |
| 369 | Mean Spectral Bandwidth (Hz)__pct32 | 2 |
| 370 | MFCC_4__t2 | 2 |
| 371 | Duration (s)__r32 | 2 |
| 372 | Mean Spectral Roll-off (Hz, 85%)__pct32 | 2 |
| 373 | Mean Spectral Roll-off (Hz, 85%)__r32 | 2 |
| 374 | Contrast_1 (dB)__t2 | 2 |
| 375 | Contrast_2 (dB)__pct21 | 2 |
| 376 | Contrast_2 (dB)__t2 | 2 |
| 377 | MFCC_10__logd32 | 2 |
| 378 | MFCC_11__t1 | 1 |
| 379 | Duration (s)__log_t1 | 1 |
| 380 | MFCC_8__t3 | 1 |
| 381 | MFCC_3__pct32 | 1 |
| 382 | Mean Spectral Centroid (Hz)__mean3 | 1 |
| 383 | Contrast_5 (dB)__std3 | 1 |
| 384 | Energy__d21 | 1 |
| 385 | Mean Amplitude__mean3 | 1 |
| 386 | Duration (s)__pct32 | 1 |
| 387 | Contrast_4 (dB)__log_t2 | 1 |
| 388 | Mean Spectral Flatness__mean3 | 1 |
| 389 | MFCC_1__pct21 | 0 |
| 390 | MFCC_2__logd32 | 0 |
| 391 | Contrast_3 (dB)__logd32 | 0 |
| 392 | MFCC_3__log_t2 | 0 |
| 393 | MFCC_6__t1 | 0 |
| 394 | MFCC_8__t1 | 0 |
| 395 | MFCC_13__t1 | 0 |
| 396 | Contrast_2 (dB)__logd21 | 0 |
| 397 | Contrast_2 (dB)__slope3 | 0 |
| 398 | MFCC_7__log_t2 | 0 |
| 399 | Duration (s)__log_t2 | 0 |
| 400 | MFCC_7__median3 | 0 |
| 401 | MFCC_9__log_t3 | 0 |
| 402 | MFCC_9__logd21 | 0 |
| 403 | MFCC_13__log_t2 | 0 |
| 404 | MFCC_10__t2 | 0 |
| 405 | MFCC_7__mean3 | 0 |
| 406 | MFCC_10__log_t3 | 0 |
| 407 | MFCC_10__log_t2 | 0 |
| 408 | MFCC_13__log_t1 | 0 |
| 409 | MFCC_13__logd21 | 0 |
| 410 | MFCC_13__logd32 | 0 |
| 411 | MFCC_7__d32 | 0 |
| 412 | Mean Amplitude__median3 | 0 |
| 413 | MFCC_7__t3 | 0 |
| 414 | MFCC_10__log_t1 | 0 |
| 415 | Duration (s)__log_t3 | 0 |
| 416 | MFCC_9__log_t2 | 0 |
| 417 | MFCC_9__pct21 | 0 |
| 418 | MFCC_7__log_t3 | 0 |
| 419 | MFCC_8__pct32 | 0 |
| 420 | MFCC_11__log_t1 | 0 |
| 421 | Duration (s)__mean3 | 0 |
| 422 | MFCC_13__d32 | 0 |
| 423 | Duration (s)__median3 | 0 |
| 424 | MFCC_11__mean3 | 0 |
| 425 | MFCC_11__log_t3 | 0 |
| 426 | MFCC_11__d21 | 0 |
| 427 | MFCC_8__log_t1 | 0 |
| 428 | MFCC_12__pct32 | 0 |
| 429 | MFCC_8__log_t3 | 0 |
| 430 | MFCC_7__pct21 | 0 |
| 431 | MFCC_8__logd32 | 0 |
| 432 | Duration (s)__logd32 | 0 |
| 433 | MFCC_12__log_t3 | 0 |
| 434 | MFCC_9__d21 | 0 |
| 435 | MFCC_12__log_t2 | 0 |
| 436 | Mean Amplitude__d21 | 0 |
| 437 | Mean Spectral Centroid (Hz)__logd32 | 0 |
| 438 | MFCC_6__logd32 | 0 |
| 439 | Peaks Detected__logd21 | 0 |
| 440 | Mean Spectral Centroid (Hz)__t2 | 0 |
| 441 | Mean Spectral Flatness__r21 | 0 |
| 442 | Mean Spectral Flatness__log_t1 | 0 |
| 443 | Mean Spectral Flatness__log_t2 | 0 |
| 444 | Mean Spectral Flatness__log_t3 | 0 |
| 445 | Mean Spectral Flatness__logd32 | 0 |
| 446 | Peaks Detected__slope3 | 0 |
| 447 | Peaks Detected__logd32 | 0 |
| 448 | Contrast_1 (dB)__range3 | 0 |
| 449 | Contrast_1 (dB)__pct21 | 0 |
| 450 | Contrast_1 (dB)__pct32 | 0 |
| 451 | Contrast_1 (dB)__log_t1 | 0 |
| 452 | Contrast_1 (dB)__log_t2 | 0 |
| 453 | Contrast_1 (dB)__log_t3 | 0 |
| 454 | Peaks Detected__log_t3 | 0 |
| 455 | Contrast_4 (dB)__log_t3 | 0 |
| 456 | Peaks Detected__log_t2 | 0 |
| 457 | Contrast_2 (dB)__log_t1 | 0 |
| 458 | Contrast_2 (dB)__log_t2 | 0 |
| 459 | Contrast_2 (dB)__log_t3 | 0 |
| 460 | Peaks Detected__log_t1 | 0 |
| 461 | Peaks Detected__pct32 | 0 |
| 462 | Peaks Detected__r32 | 0 |
| 463 | Contrast_3 (dB)__pct21 | 0 |
| 464 | Contrast_3 (dB)__log_t1 | 0 |
| 465 | Contrast_3 (dB)__log_t3 | 0 |
| 466 | Peaks Detected__pct21 | 0 |
| 467 | Peaks Detected__r21 | 0 |
| 468 | Contrast_4 (dB)__pct21 | 0 |
| 469 | Peaks Detected__range3 | 0 |
| 470 | Mean Spectral Flatness__t2 | 0 |
| 471 | Mean Spectral Roll-off (Hz, 85%)__logd21 | 0 |
| 472 | Mean Spectral Roll-off (Hz, 85%)__log_t3 | 0 |
| 473 | Mean Spectral Roll-off (Hz, 85%)__log_t2 | 0 |
| 474 | Mean Spectral Centroid (Hz)__log_t3 | 0 |
| 475 | Mean Spectral Centroid (Hz)__log_t2 | 0 |
| 476 | Mean Spectral Centroid (Hz)__log_t1 | 0 |
| 477 | Mean Spectral Centroid (Hz)__pct32 | 0 |
| 478 | Mean Zero Crossing Rate__pct32 | 0 |
| 479 | Mean Zero Crossing Rate__log_t1 | 0 |
| 480 | Mean Zero Crossing Rate__logd21 | 0 |
| 481 | Mean Spectral Centroid (Hz)__pct21 | 0 |
| 482 | Mean RMS__t1 | 0 |
| 483 | Mean RMS__t3 | 0 |
| 484 | Mean RMS__std3 | 0 |
| 485 | Mean RMS__median3 | 0 |
| 486 | Mean RMS__pct21 | 0 |
| 487 | Mean RMS__pct32 | 0 |
| 488 | Mean RMS__log_t1 | 0 |
| 489 | Mean RMS__log_t2 | 0 |
| 490 | Mean RMS__log_t3 | 0 |
| 491 | Mean RMS__logd21 | 0 |
| 492 | Mean Spectral Bandwidth (Hz)__t3 | 0 |
| 493 | Mean Spectral Centroid (Hz)__median3 | 0 |
| 494 | Mean Spectral Bandwidth (Hz)__mean3 | 0 |
| 495 | Mean Spectral Bandwidth (Hz)__pct21 | 0 |
| 496 | Mean Spectral Bandwidth (Hz)__log_t2 | 0 |
| 497 | Mean Spectral Bandwidth (Hz)__log_t3 | 0 |
| 498 | Mean Spectral Bandwidth (Hz)__logd21 | 0 |
| 499 | Mean Spectral Bandwidth (Hz)__logd32 | 0 |
| 500 | Mean Spectral Roll-off (Hz, 85%)__median3 | 0 |
| 501 | Mean Spectral Roll-off (Hz, 85%)__pct21 | 0 |
| 502 | Mean Spectral Roll-off (Hz, 85%)__log_t1 | 0 |
| 503 | Contrast_4 (dB)__log_t1 | 0 |
| 504 | Contrast_4 (dB)__logd32 | 0 |
| 505 | MFCC_6__log_t3 | 0 |
| 506 | MFCC_4__r21 | 0 |
| 507 | Energy__log_t3 | 0 |
| 508 | MFCC_2__t2 | 0 |
| 509 | Energy__log_t2 | 0 |
| 510 | MFCC_2__median3 | 0 |
| 511 | Energy__pct32 | 0 |
| 512 | Energy__r32 | 0 |
| 513 | MFCC_2__log_t1 | 0 |
| 514 | MFCC_2__log_t2 | 0 |
| 515 | MFCC_3__mean3 | 0 |
| 516 | Energy__median3 | 0 |
| 517 | Energy__std3 | 0 |
| 518 | MFCC_3__log_t1 | 0 |
| 519 | MFCC_3__log_t3 | 0 |
| 520 | MFCC_4__t1 | 0 |
| 521 | MFCC_4__log_t1 | 0 |
| 522 | Peaks Detected__median3 | 0 |
| 523 | MFCC_4__log_t2 | 0 |
| 524 | MFCC_4__log_t3 | 0 |
| 525 | MFCC_4__logd32 | 0 |
| 526 | Mean Amplitude__log_t3 | 0 |
| 527 | Mean Amplitude__pct32 | 0 |
| 528 | Mean Amplitude__r32 | 0 |
| 529 | MFCC_5__log_t3 | 0 |
| 530 | MFCC_5__logd32 | 0 |
| 531 | MFCC_6__t2 | 0 |
| 532 | MFCC_6__mean3 | 0 |
| 533 | Mean Amplitude__pct21 | 0 |
| 534 | MFCC_6__pct32 | 0 |
| 535 | MFCC_6__log_t1 | 0 |
| 536 | MFCC_6__log_t2 | 0 |
| 537 | MFCC_1__logd21 | 0 |
| 538 | MFCC_1__log_t3 | 0 |
| 539 | MFCC_1__log_t2 | 0 |
| 540 | MFCC_1__log_t1 | 0 |
| 541 | Peaks Detected__std3 | 0 |
| 542 | Peaks Detected__mean3 | 0 |
| 543 | Peaks Detected__d32 | 0 |
| 544 | Contrast_5 (dB)__pct21 | 0 |
| 545 | Contrast_5 (dB)__log_t1 | 0 |
| 546 | Contrast_5 (dB)__log_t2 | 0 |
| 547 | Contrast_5 (dB)__logd21 | 0 |
| 548 | Peaks Detected__d21 | 0 |
| 549 | Contrast_6 (dB)__pct21 | 0 |
| 550 | Contrast_6 (dB)__pct32 | 0 |
| 551 | Contrast_6 (dB)__log_t1 | 0 |
| 552 | Contrast_6 (dB)__log_t2 | 0 |
| 553 | Contrast_6 (dB)__log_t3 | 0 |
| 554 | Contrast_6 (dB)__logd21 | 0 |
| 555 | Contrast_6 (dB)__logd32 | 0 |
| 556 | Peaks Detected__t3 | 0 |
| 557 | Peaks Detected__t2 | 0 |
| 558 | Peaks Detected__t1 | 0 |
| 559 | Contrast_7 (dB)__pct21 | 0 |
| 560 | Contrast_7 (dB)__pct32 | 0 |
| 561 | Contrast_7 (dB)__log_t2 | 0 |
| 562 | Contrast_7 (dB)__log_t3 | 0 |
| 563 | Contrast_7 (dB)__logd21 | 0 |
| 564 | Contrast_7 (dB)__logd32 | 0 |
| 565 | Energy__logd32 | 0 |
| 566 | MFCC_1__mean3 | 0 |
| 567 | MFCC_1__median3 | 0 |
| 568 | Mean Spectral Centroid (Hz)__logd21 | 0 |
| 569 | Energy__logd21 | 0 |
| 570 | MFCC_1__r21 | 0 |
