# Supplementary material for: Acoustic cues into a surgeon-assist physical AI for detecting bone penetration during spinal surgery
Source: Sci Rep. 2026 Apr 19;16:18113. doi: 10.1038/s41598-026-48857-w (PMC13254284; doi:10.1038/s41598-026-48857-w)
Supplement: Supplementary file 4 — Supplementary Material 4 [file 41598_2026_48857_MOESM4_ESM.docx]

**Model Training and Evaluation Code**

**Overview**

This supplementary file provides the complete Python code used for model training, validation, operating threshold determination, and independent test evaluation in this study. The code implements a reproducible machine learning pipeline designed to detect cortical bone penetration from intraoperative percussion sounds while preventing information leakage at the recording level.

**Software requirements**

The analysis was conducted using the following software environment:

- **Python**: 3.10.14
- **LightGBM**: 4.5.0
- **scikit-learn**: 1.5.2
- **pandas**: 2.2.2
- **NumPy**: 2.1.3

All analyses are fully deterministic when executed with the specified random seed (RANDOM_STATE = 42).

**Input data**

The code expects a single input file:

- **File name**: window3_dataset_plus.csv

This file contains acoustic features extracted from intraoperative chisel percussion sounds using a three-strike sliding window approach.

**Required columns**

The following columns must be present in the input dataset:

- ID: Patient identifier
- Recording: Recording identifier
- grp_key: Unique identifier for each recording session (used for group-based splitting)
- y: Binary outcome label
  - 1 = bone penetration
  - 0 = non-penetration
- Additional columns corresponding to acoustic features (all remaining columns are treated as input features)

All windows derived from the same recording must share the same grp_key value.

**Data splitting and validation strategy**

To prevent information leakage, all data splits are performed at the **recording level** using grp_key:

1. **Independent hold-out test set**
   - 10% of recordings are reserved using GroupShuffleSplit
   - The test set is not used for model selection or threshold determination
2. **Cross-validation on the training set**
   - GroupKFold cross-validation (up to 5 folds) is applied
   - All windows from a given recording appear in only one fold

**Model training**

A gradient boosting classifier implemented with LightGBM is trained using an expanded acoustic feature set (570 features per three-strike window). Class imbalance is addressed by applying fold-specific positive class weighting (scale_pos_weight = n_negative / n_positive). Early stopping is used during cross-validation to determine the optimal number of boosting iterations.

**Operating threshold determination**

The operating threshold for binary classification is determined exclusively from **out-of-fold (OOF) predictions** on the training set. The threshold is selected as the maximum probability value that achieves a predefined target sensitivity (recall ≥ 0.80), reflecting clinical safety requirements. Importantly, the independent test set is not used in any step of threshold selection.

**Output files**

Running the script generates the following output files:

- cv_results_3hit_plus_pr.csv: Cross-validation performance summary
- holdout_test_metrics_3hit_plus_pr.csv: Performance metrics on the independent test set
- holdout_pred_3hit_plus_pr.csv: Test set predictions with probabilities
- feature_importances_3hit_plus_pr.csv: Feature importance based on gain
- internal_class_balance_3hit_plus_pr.csv: Class distribution in internal splits
- Group composition files documenting recording-level splits

**Reproducibility**

All steps of model training, threshold determination, and evaluation are fully specified in the accompanying code. By fixing the random seed and enforcing recording-level data partitioning, the pipeline enables transparent and reproducible validation of the reported results.

import json

import numpy as np

import pandas as pd

from sklearn.model_selection import GroupKFold, GroupShuffleSplit

from sklearn.metrics import (

roc_auc_score, average_precision_score,

accuracy_score, f1_score, precision_score, recall_score,

confusion_matrix, precision_recall_curve

)

import lightgbm as lgb

# -------------------

# Configuration

# -------------------

DATA_PATH = "window3_dataset_plus.csv"

TEST_FRACTION = 0.10

INNER_FOLDS = 5

RANDOM_STATE = 42

PRIMARY_METRIC = "average_precision" # PR-AUC

TARGET_SENS = 0.80 # Operating point: target sensitivity (recall) = 0.80

# Parameter grid (extend as needed)

PARAM_GRID = [

{"num_leaves": 31, "min_data_in_leaf": 20, "feature_fraction": 0.8, "bagging_fraction": 0.8},

{"num_leaves": 63, "min_data_in_leaf": 30, "feature_fraction": 0.9, "bagging_fraction": 0.8},

]

# -------------------

# Utility functions

# -------------------

def mean_std(xs):

return float(np.mean(xs)), float(np.std(xs))

def summarize_metrics(y_true, prob, threshold):

"""

Summarize metrics (threshold-based classification + AUC/PR).

"""

y_true = np.asarray(y_true).astype(int)

prob = np.asarray(prob).astype(float)

pred = (prob >= threshold).astype(int)

# Confusion matrix (guard against single-class cases)

try:

tn, fp, fn, tp = confusion_matrix(y_true, pred, labels=[0, 1]).ravel()

except Exception:

# If an exception occurs, fill with zeros

tn = fp = fn = tp = 0

sens = tp / (tp + fn) if (tp + fn) > 0 else 0.0

spec = tn / (tn + fp) if (tn + fp) > 0 else 0.0

# Compute AUC/PR only when both classes are present

auc = float(roc_auc_score(y_true, prob)) if len(np.unique(y_true)) > 1 else float("nan")

pr = float(average_precision_score(y_true, prob)) if len(np.unique(y_true)) > 1 else float("nan")

return {

"AUC": auc,

"PR": pr,

"ACC": float(accuracy_score(y_true, pred)),

"F1": float(f1_score(y_true, pred, zero_division=0)),

"PREC": float(precision_score(y_true, pred, zero_division=0)),

"REC": float(recall_score(y_true, pred, zero_division=0)),

"SENS": float(sens),

"SPEC": float(spec),

"threshold": float(threshold),

"tp": int(tp), "fp": int(fp), "tn": int(tn), "fn": int(fn),

}

def threshold_at_target_sensitivity(y_true, prob, target_sens=0.80):

"""

Return the *maximum threshold* that satisfies sensitivity >= target_sens.

- Intention: preserve sensitivity while raising the threshold as much as possible

to reduce precision collapse.

If the target is not achievable:

- Fall back toward maximal sensitivity (i.e., the minimum threshold).

Returns:

tuple: (threshold, achieved_sensitivity, is_achievable)

"""

y_true = np.asarray(y_true).astype(int)

prob = np.asarray(prob).astype(float)

pos = int((y_true == 1).sum())

if pos == 0:

return 0.50, 0.0, False # No positives -> cannot design a threshold

prec, rec, thr = precision_recall_curve(y_true, prob)

if len(thr) == 0:

return 0.50, 0.0, False

# For thr[i], the corresponding rec/prec are rec[i+1], prec[i+1]

rec_for_thr = rec[1:]

ok = np.where(rec_for_thr >= target_sens)[0]

if len(ok) == 0:

# Target not achievable: return the minimum threshold (closest to max sensitivity),

# but mark as not achievable

best_thr = float(np.min(thr))

achieved_sens = float(np.max(rec_for_thr)) if len(rec_for_thr) > 0 else 0.0

return best_thr, achieved_sens, False

# Among thresholds satisfying the condition, take the maximum threshold

best_idx = ok[np.argmax(thr[ok])]

best_thr = float(thr[best_idx])

achieved_sens = float(rec_for_thr[best_idx])

return best_thr, achieved_sens, True

def save_group_lists(df, te_idx, tr_idx, grp_map, suffix="3hit_plus"):

test_groups_df = df.iloc[te_idx, :][["grp_key"]].drop_duplicates().join(grp_map, on="grp_key").reset_index(drop=True)

test_sizes = (

df.iloc[te_idx, :]["grp_key"].value_counts().rename_axis("grp_key").reset_index(name="n_rows")

.merge(test_groups_df, on="grp_key", how="left")[["ID","Recording","grp_key","n_rows"]]

)

test_groups_df.to_csv(f"holdout_test_groups_{suffix}.csv", index=False, encoding="utf-8-sig")

test_sizes.to_csv(f"holdout_test_group_sizes_{suffix}.csv", index=False, encoding="utf-8-sig")

train_groups_df = df.iloc[tr_idx, :][["grp_key"]].drop_duplicates().join(grp_map, on="grp_key").reset_index(drop=True)

train_sizes = (

df.iloc[tr_idx, :]["grp_key"].value_counts().rename_axis("grp_key").reset_index(name="n_rows")

.merge(train_groups_df, on="grp_key", how="left")[["ID","Recording","grp_key","n_rows"]]

)

train_groups_df.to_csv(f"holdout_train_groups_{suffix}.csv", index=False, encoding="utf-8-sig")

train_sizes.to_csv(f"holdout_train_group_sizes_{suffix}.csv", index=False, encoding="utf-8-sig")

def save_internal_lists(groups_tr2, groups_va2, grp_map):

inner_train_df = pd.DataFrame({"grp_key": pd.unique(groups_tr2)}).join(grp_map, on="grp_key").reset_index(drop=True)

inner_valid_df = pd.DataFrame({"grp_key": pd.unique(groups_va2)}).join(grp_map, on="grp_key").reset_index(drop=True)

inner_train_sizes = (

pd.Series(groups_tr2).value_counts().rename_axis("grp_key").reset_index(name="n_rows")

.merge(inner_train_df, on="grp_key", how="left")[["ID","Recording","grp_key","n_rows"]]

)

inner_valid_sizes = (

pd.Series(groups_va2).value_counts().rename_axis("grp_key").reset_index(name="n_rows")

.merge(inner_valid_df, on="grp_key", how="left")[["ID","Recording","grp_key","n_rows"]]

)

inner_train_df.to_csv("internal_train_groups_3hit_plus.csv", index=False, encoding="utf-8-sig")

inner_valid_df.to_csv("internal_valid_groups_3hit_plus.csv", index=False, encoding="utf-8-sig")

inner_train_sizes.to_csv("internal_train_group_sizes_3hit_plus.csv", index=False, encoding="utf-8-sig")

inner_valid_sizes.to_csv("internal_valid_group_sizes_3hit_plus.csv", index=False, encoding="utf-8-sig")

# -------------------

# Load data

# -------------------

df = pd.read_csv(DATA_PATH)

non_feature_cols = ["ID","Recording","StartSeq","MidSeq","EndSeq","Label","y","grp_key"]

for c in non_feature_cols:

if c not in df.columns:

raise ValueError(f"Expected column '{c}' not found. Columns head: {list(df.columns)[:25]}")

feat_cols = [c for c in df.columns if c not in non_feature_cols]

X_all = df[feat_cols].values

y_all = df["y"].astype(int).values

groups_all = df["grp_key"].astype(str).values

grp_map = df[["grp_key","ID","Recording"]].drop_duplicates().set_index("grp_key")

print(f"[Dataset] Total rows={len(df)}, Features={len(feat_cols)}")

print(f"[Dataset] Total groups(recordings)={len(np.unique(groups_all))}")

print(f"[Dataset] Overall positive rate={y_all.mean():.4f} ({(y_all==1).sum()}/{len(y_all)})")

# -------------------

# 10% hold-out at the recording level (stable split)

# -------------------

unique_groups = np.unique(groups_all)

n_groups = len(unique_groups)

if n_groups < 4:

raise ValueError(f"Too few recordings(grp_key)={n_groups}. Need at least ~4 for holdout+CV stability.")

gss = GroupShuffleSplit(n_splits=1, test_size=TEST_FRACTION, random_state=RANDOM_STATE)

tr_idx, te_idx = next(gss.split(X_all, y_all, groups_all))

X_train, X_test = X_all[tr_idx], X_all[te_idx]

y_train, y_test = y_all[tr_idx], y_all[te_idx]

groups_train = groups_all[tr_idx]

groups_test = groups_all[te_idx]

print(f"\n[Hold-out Split]")

print(f" Train: rows={len(tr_idx)}, groups={len(np.unique(groups_train))}, pos%={y_train.mean():.4f}")

print(f" Test: rows={len(te_idx)}, groups={len(np.unique(groups_test))}, pos%={y_test.mean():.4f}")

# Save recording lists

save_group_lists(df, te_idx, tr_idx, grp_map, suffix="3hit_plus")

# -------------------

# Inner CV (GroupKFold) — primary metric: PR-AUC

# - Automatically adjust n_splits (avoid failure with few recordings)

# - Determine the operating threshold from OOF predictions (target sensitivity=0.8)

# -------------------

n_train_groups = len(np.unique(groups_train))

inner_splits = min(INNER_FOLDS, n_train_groups)

if inner_splits < 2:

raise ValueError(f"Too few train recordings={n_train_groups} for inner CV. Need at least 2.")

print(f"\n[Cross-Validation Setup]")

print(f" Inner splits (GroupKFold): {inner_splits}")

print(f" Parameter grid size: {len(PARAM_GRID)}")

gkf = GroupKFold(n_splits=inner_splits)

cv_rows = []

for param_idx, params in enumerate(PARAM_GRID, start=1):

print(f"\n[CV {param_idx}/{len(PARAM_GRID)}] Testing params: {params}")

prs, aucs, accs, f1s, pres, recs = [], [], [], [], [], []

best_iters = []

valid_folds = 0 # Count folds where both classes are present

# Accumulate OOF probabilities and determine the threshold once at the end

oof_prob = np.full(len(y_train), np.nan, dtype=float)

for fold, (tr_i, va_i) in enumerate(gkf.split(X_train, y_train, groups_train), start=1):

Xtr, Xva = X_train[tr_i], X_train[va_i]

ytr, yva = y_train[tr_i], y_train[va_i]

# Imbalance handling per fold

pos = int((ytr == 1).sum())

neg = int((ytr == 0).sum())

if pos == 0:

raise ValueError(f"Fold {fold}: No positive samples in training set. Check data split.")

if neg == 0:

raise ValueError(f"Fold {fold}: No negative samples in training set. Check data split.")

spw = neg / pos

clf = lgb.LGBMClassifier(

objective="binary",

metric=PRIMARY_METRIC,

learning_rate=0.05,

n_estimators=1500,

num_leaves=params["num_leaves"],

min_data_in_leaf=params["min_data_in_leaf"],

feature_fraction=params["feature_fraction"],

bagging_fraction=params["bagging_fraction"],

bagging_freq=1,

max_depth=-1,

n_jobs=-1,

random_state=RANDOM_STATE,

scale_pos_weight=spw

)

clf.fit(

Xtr, ytr,

eval_set=[(Xva, yva)],

eval_metric=PRIMARY_METRIC,

callbacks=[lgb.early_stopping(stopping_rounds=100, verbose=False)]

)

prob_va = clf.predict_proba(Xva, num_iteration=clf.best_iteration_)[:, 1]

oof_prob[va_i] = prob_va

best_iters.append(int(clf.best_iteration_))

# Fold-level PR/AUC (threshold-independent primary metrics)

if len(np.unique(yva)) > 1:

prs.append(float(average_precision_score(yva, prob_va)))

aucs.append(float(roc_auc_score(yva, prob_va)))

valid_folds += 1

else:

prs.append(float("nan"))

aucs.append(float("nan"))

print(f" Warning: Fold {fold} has only one class in validation set")

# Temporary evaluation at threshold=0.5 (final operating threshold is determined from OOF)

pred05 = (prob_va >= 0.5).astype(int)

accs.append(float(accuracy_score(yva, pred05)))

f1s.append(float(f1_score(yva, pred05, zero_division=0)))

pres.append(float(precision_score(yva, pred05, zero_division=0)))

recs.append(float(recall_score(yva, pred05, zero_division=0)))

# Check that all OOF predictions are filled

if np.isnan(oof_prob).any():

raise RuntimeError("OOF probabilities contain NaN. Check GroupKFold split / indexing.")

# === Operating threshold: determined from full OOF (without touching the test set) ===

op_thr, achieved_sens, is_achievable = threshold_at_target_sensitivity(

y_train, oof_prob, target_sens=TARGET_SENS

)

if not is_achievable:

print(f" WARNING: Target sensitivity {TARGET_SENS:.2f} not achievable. Best achieved: {achieved_sens:.4f}")

# Reference: operating-point performance on OOF (approx. internal generalization)

oof_metrics = summarize_metrics(y_train, oof_prob, threshold=op_thr)

res = {

"Params": json.dumps(params, ensure_ascii=False),

"PR_mean": float(np.nanmean(prs)), "PR_std": float(np.nanstd(prs)),

"AUC_mean": float(np.nanmean(aucs)), "AUC_std": float(np.nanstd(aucs)),

"ACC@0.5_mean": mean_std(accs)[0], "ACC@0.5_std": mean_std(accs)[1],

"F1@0.5_mean": mean_std(f1s)[0], "F1@0.5_std": mean_std(f1s)[1],

"PREC@0.5_mean": mean_std(pres)[0],"PREC@0.5_std": mean_std(pres)[1],

"REC@0.5_mean": mean_std(recs)[0], "REC@0.5_std": mean_std(recs)[1],

"op_thr_targetSens": float(TARGET_SENS),

"op_thr_oof": float(op_thr),

"op_thr_achieved_sens": float(achieved_sens),

"op_thr_achievable": bool(is_achievable),

"OOF_SENS": float(oof_metrics["SENS"]),

"OOF_SPEC": float(oof_metrics["SPEC"]),

"OOF_PREC": float(oof_metrics["PREC"]),

"OOF_REC": float(oof_metrics["REC"]),

"OOF_F1": float(oof_metrics["F1"]),

"cv_best_iter_mean": float(np.mean(best_iters)),

"cv_best_iter_std": float(np.std(best_iters)),

"n_train_rows": int(len(y_train)),

"n_train_groups": int(n_train_groups),

"n_features": int(X_train.shape[1]),

"inner_splits_used": int(inner_splits),

"valid_folds": int(valid_folds), # Number of folds where both classes are present

}

cv_rows.append(res)

print(f" PR-AUC: {res['PR_mean']:.4f}±{res['PR_std']:.4f}")

print(f" OOF threshold: {op_thr:.4f} (achieved sens: {achieved_sens:.4f})")

cv_df = pd.DataFrame(cv_rows).sort_values("PR_mean", ascending=False).reset_index(drop=True)

cv_df.to_csv("cv_results_3hit_plus_pr.csv", index=False, encoding="utf-8-sig")

print("\n" + "="*80)

print("[CV RESULTS — primary=PR-AUC]")

print("="*80)

print(cv_df[["Params","PR_mean","PR_std","AUC_mean","op_thr_oof","op_thr_achieved_sens","OOF_SENS","valid_folds"]].to_string(index=False))

best_params = json.loads(cv_df.loc[0, "Params"])

cv_best_iter = int(round(cv_df.loc[0, "cv_best_iter_mean"]))

op_threshold = float(cv_df.loc[0, "op_thr_oof"])

op_achievable = bool(cv_df.loc[0, "op_thr_achievable"])

print(f"\n[Best Model Selected]")

print(f" Parameters: {best_params}")

print(f" CV average iterations: {cv_best_iter}")

print(f" Operating threshold: {op_threshold:.6f}")

print(f" Target sensitivity achievable: {op_achievable}")

# -------------------

# Final training (full train) + 10% internal validation (recording-level)

# * Train with a fixed number of iterations based on CV (no early stopping)

# * Use the operating threshold op_threshold fixed above (do not touch the test set)

# -------------------

print(f"\n[Final Model Training]")

gss_inner = GroupShuffleSplit(n_splits=1, test_size=0.10, random_state=RANDOM_STATE)

tr_i2, va_i2 = next(gss_inner.split(X_train, y_train, groups_train))

Xtr2, Xva2 = X_train[tr_i2], X_train[va_i2]

ytr2, yva2 = y_train[tr_i2], y_train[va_i2]

groups_tr2 = groups_train[tr_i2]

groups_va2 = groups_train[va_i2]

print(f" Final train: rows={len(tr_i2)}, groups={len(np.unique(groups_tr2))}, pos%={ytr2.mean():.4f}")

print(f" Final valid: rows={len(va_i2)}, groups={len(np.unique(groups_va2))}, pos%={yva2.mean():.4f}")

# Validate class balance

if ytr2.sum() == 0:

raise ValueError("Final training set has no positive samples. Adjust split.")

if (ytr2 == 0).sum() == 0:

raise ValueError("Final training set has no negative samples. Adjust split.")

save_internal_lists(groups_tr2, groups_va2, grp_map)

# Imbalance handling on the final training split

pos = int((ytr2 == 1).sum())

neg = int((ytr2 == 0).sum())

spw = neg / pos

# Train with a fixed number of iterations based on CV (no early stopping)

final_clf = lgb.LGBMClassifier(

objective="binary",

metric=PRIMARY_METRIC,

learning_rate=0.05,

n_estimators=cv_best_iter, # Use the CV mean optimal iteration count

num_leaves=best_params["num_leaves"],

min_data_in_leaf=best_params["min_data_in_leaf"],

feature_fraction=best_params["feature_fraction"],

bagging_fraction=best_params["bagging_fraction"],

bagging_freq=1,

max_depth=-1,

n_jobs=-1,

random_state=RANDOM_STATE,

scale_pos_weight=spw

)

# The validation set is for monitoring only (not used for early stopping)

final_clf.fit(Xtr2, ytr2, eval_set=[(Xva2, yva2)], eval_metric=PRIMARY_METRIC)

print(f" Model trained with n_estimators={cv_best_iter} (no early stopping)")

# -------------------

# Evaluate on the independent test set (PR-AUC + operating threshold op_threshold)

# -------------------

print(f"\n[Hold-out Test Evaluation]")

prob_te = final_clf.predict_proba(X_test)[:, 1]

test_metrics = summarize_metrics(y_test, prob_te, threshold=op_threshold)

pd.DataFrame([test_metrics]).to_csv("holdout_test_metrics_3hit_plus_pr.csv", index=False, encoding="utf-8-sig")

print("="*80)

print("[HOLD-OUT TEST METRICS]")

print("="*80)

print(pd.DataFrame([test_metrics]).to_string(index=False))

# Prediction details

te_df = df.iloc[te_idx, :].copy()

te_df["prob"] = prob_te

te_df["pred"] = (prob_te >= op_threshold).astype(int)

te_df.to_csv("holdout_pred_3hit_plus_pr.csv", index=False, encoding="utf-8-sig")

# Feature importances

importances = pd.DataFrame({

"feature": feat_cols,

"importance": final_clf.booster_.feature_importance(importance_type="gain")

}).sort_values("importance", ascending=False)

importances.to_csv("feature_importances_3hit_plus_pr.csv", index=False, encoding="utf-8-sig")

print(f"\n[Top 10 Important Features]")

print(importances.head(10).to_string(index=False))

# Class distribution in internal train/valid splits (reference)

df_train_pool = df.iloc[tr_idx, :].copy()

inner_train_df = df_train_pool.iloc[tr_i2, :].copy()

inner_valid_df = df_train_pool.iloc[va_i2, :].copy()

def count_p_np(frame):

return {

"n_rows": int(len(frame)),

"n_p(y=1)": int((frame["y"] == 1).sum()),

"n_np(y=0)": int((frame["y"] == 0).sum()),

"pos_rate(p%)": float((frame["y"] == 1).mean())

}

summary_df = pd.DataFrame([

{"split": "internal_train", **count_p_np(inner_train_df)},

{"split": "internal_valid", **count_p_np(inner_valid_df)}

])

summary_df.to_csv("internal_class_balance_3hit_plus_pr.csv", index=False, encoding="utf-8-sig")

print(f"\n[Internal Split Class Balance]")

print(summary_df.to_string(index=False))

print("\n" + "="*80)

print("[Saved Files]")

print("="*80)

for p in [

"cv_results_3hit_plus_pr.csv",

"holdout_test_metrics_3hit_plus_pr.csv",

"holdout_pred_3hit_plus_pr.csv",

"feature_importances_3hit_plus_pr.csv",

"internal_class_balance_3hit_plus_pr.csv",

"holdout_test_groups_3hit_plus.csv",

"holdout_test_group_sizes_3hit_plus.csv",

"holdout_train_groups_3hit_plus.csv",

"holdout_train_group_sizes_3hit_plus.csv",

"internal_train_groups_3hit_plus.csv",

"internal_valid_groups_3hit_plus.csv",

"internal_train_group_sizes_3hit_plus.csv",

"internal_valid_group_sizes_3hit_plus.csv",

]:

print(f" ✓ {p}")

print("\n" + "="*80)

print("All done (3-hit+extra features, primary metric = PR-AUC, fixed iterations)")

print("="*80)
