# Supplementary material for: Acoustic cues into a surgeon-assist physical AI for detecting bone penetration during spinal surgery
Source: Sci Rep. 2026 Apr 19;16:18113. doi: 10.1038/s41598-026-48857-w (PMC13254284; doi:10.1038/s41598-026-48857-w)
Supplement: Supplementary file 5 — Supplementary Material 5 [file 41598_2026_48857_MOESM5_ESM.docx]

The STARD-AI (Standards for Reporting of Diagnostic accuracy-Artificial Intelligence) checklist

| **Section and Topic** | **No.** | **STARD-AI Item** | **Reported on page** |
| --- | --- | --- | --- |
| **Title or abstract** | | | |
|  | 1 | Identification as a study reporting AI-centred diagnostic accuracy and reporting at least one measure of accuracy within title or abstract | abstract |
| **Abstract** | | | |
|  | 2 | Structured summary of study design, methods, results and conclusions (for specific guidance, please see STARD for Abstracts) | abstract |
| **Introduction** | | | |
|  | 3 | Scientific and clinical background, including the intended use of the index test, whether it is novel or an established index test, and its integration into an existing or new workflow, if applicable | Introduction; paragraph1 |
|  | 4 | Study objectives and hypotheses | Introduction; paragraph4 |
| **Methods** | | | |
| Study design | 5 | Whether data collection was planned before the index test and reference standard were performed (prospective study) or after (retrospective study) | Methods; Data collection and labeling; paragraph1 |
| Ethics | 6 | Formal approval from an ethics committee. If not required, justify why | Title page |
| Participants | 7 | Eligibility criteria: listing separate inclusion and exclusion criteria in the order that they are applied at both participant level and data level | Methods; Data collection and labeling; paragraph1 |
|  | 8 | On what basis potentially eligible participants were identified (such as symptoms, results from previous tests, inclusion in registry) | Methods; Data collection and labeling; paragraph1 |
|  | 9 | Where and when potentially eligible participants were identified (setting, location, and dates) | Methods Data collection and labeling; paragraph1 |
|  | 10 | Whether participants formed a consecutive, random, or convenience series | Methods; Data collection and labeling; paragraph1 |
| Dataset | 11 | Source of the data and whether it has been routinely collected, specifically collected for the purpose of the study or acquired from an open-source repository | Methods; Data collection and labeling; paragraph1-2 |
|  | 12 | Who undertook the annotations for the dataset (including experience levels and background) and how (within the same clinical context or in a post-hoc fashion), if applicable | Methods; Data collection and labeling; paragraph2 |
|  | 13 | Devices (manufacturer, model) that were used to capture data; software (with version number) used to engineer the index test, highlighting the intended use | Methods; Data collection and labeling; paragraph1  Methods; Statistical analysis; paragraph 1 |
|  | 14 | Data acquisition protocols (e.g. contrast protocol or reconstruction method for medical images) and details of data pre- processing in sufficient detail to allow replication | Methods; Data collection and labeling; paragraph1-3  Methods; Acoustic feature extraction |
| Test methods | 15a | Index test, in sufficient detail to allow replication | Methods; AI model development; paragraphs 1–3 |
|  | 15b | How the index test was developed, including any training, validation, testing and external evaluation, detailing sample sizes, when applicable | Methods; AI model development; paragraphs 1–4 |
|  | 15c | Definition of and rationale for test positivity cut-offs or result categories of the index test, distinguishing pre-specified from exploratory | Methods; AI model development; paragraph 3 |
|  | 15d | The specified end user of the index test and the level of expertise required of users | Methods; AI model development; paragraph 1 |
|  | 16a | Reference standard, in sufficient detail to allow replication | Methods; Data collection and labeling; paragraph 2 |

|  | 16b | Rationale for choosing the reference standard (if alternatives exist) | Methods; Data collection and labeling; paragraph 2 |
| --- | --- | --- | --- |
|  | 16c | Definition of and rationale for test positivity cut-offs or result categories of the reference standard, distinguishing pre-specified from exploratory | Methods; Data collection and labeling; paragraph 1–2 |
|  | 17a | Whether clinical information and reference standard results were available to the performers or readers of the index test | Methods; AI model development; paragraph 1 |
|  | 17b | Whether clinical information and index test results were available to the assessors of the reference standard | Methods; AI model development; paragraph 1 |
| Analysis | 18 | Methods for estimating or comparing measures of diagnostic accuracy | Methods; Statistical analysis; paragraph 1 |
|  | 19 | How indeterminate index test or reference standard results were handled | Methods; Data collection and labeling; paragraph 1–2 |
|  | 20 | How missing data on the index test and reference standard were handled | Methods; AI model development; paragraph 3 |
|  | 21 | Any analyses of variability in diagnostic accuracy, distinguishing pre-specified from exploratory | Methods; AI model development |
|  | 22 | Intended sample size and how it was determined |  |
|  | 23 | Details of any performance error analysis, and algorithmic bias and fairness assessments if undertaken |  |
| **Results** | | | |
| Participants and dataset | 24 | Flow of participants, using a diagram | Results; Demographics; paragraph 1 Figure 1 |
|  | 25 | Baseline demographic, clinical and technical characteristics of training, validation and test set, if applicable | Results; Demographics; paragraph 1  Results; Three-strike windows; paragraph 1 |
|  | 26a | Distribution of severity of disease in those with the target condition | Results; Demographics; paragraph 1 |
|  | 26b | Distribution of alternative diagnoses in those without the target condition | Results; Demographics; paragraph 1 |
|  | 27 | Time interval and any clinical interventions between index test and reference standard | Methods; Data collection and labeling; paragraph 2  Results |
|  | 28 | Whether the datasets represent the distribution of the target condition that one would expect from the intended use population | Results; Demographics; paragraph 1  Discussion; Clinical relevance |
|  | 29 | For external evaluation on an independent dataset, an assessment of how this differs from the training, validation and test sets | Results; Three-strike windows; paragraph 1  Results; Model performance; paragraph 2 |
| Test results | 30 | Cross tabulation of the index test results (or their distribution) by the results of the reference standard | Results; Model performance; paragraph 3 Table 3 |
|  | 31 | Estimates of diagnostic accuracy and their precision (such as 95% confidence intervals) | Results; Model performance; paragraphs 1–2  Tables 2 and 3 |
|  | 32 | Any adverse events from performing the index test or the reference standard |  |
| **Discussion** | | | |
|  | 33 | Study limitations, including sources of potential bias, statistical uncertainty, and generalisability | Discussion; Limitations; paragraphs 1–3 |
|  | 34 | Implications for practice, including the intended use and clinical role of the index test | Discussion; Clinical relevance; paragraph 1–2  Conclusion; paragraph 1–2 |
|  | 35 | Ethical considerations and adherence to ethical standards associated with the use of the index test and issues of fairness | Methods |
| **Other information** | | | |
|  | 36 | Registration number and name of registry |  |
|  | 37 | Where the full study protocol can be accessed |  |
|  | 38 | Sources of funding and other support; role of funders |  |
|  | 39 | Commercial interests, if applicable |  |
|  | 40a | Availability of datasets and code; detailing any restrictions on their reuse and repurposing |  |
|  | 40b | Whether outputs are stored, auditable and available for evaluation, if necessary |  |
