# Supplementary material for: Acoustic cues into a surgeon-assist physical AI for detecting bone penetration during spinal surgery
Source: Sci Rep. 2026 Apr 19;16:18113. doi: 10.1038/s41598-026-48857-w (PMC13254284; doi:10.1038/s41598-026-48857-w)
Supplement: Supplementary file 6 — Supplementary Material 6 [file 41598_2026_48857_MOESM6_ESM.docx]

**Supplementary. Benchmark comparison with a CNN-LSTM model**

To address the reviewer’s request for comparison with representative deep sequential models, we additionally evaluated a convolutional neural network–long short-term memory (CNN-LSTM) benchmark model using the same recording-level data split and evaluation framework as the proposed LightGBM model.

For this benchmark, each 3-hit window was represented as a sequential input composed of the 30 base acoustic features extracted from each of the three consecutive strikes (input shape: 3 × 30). We intentionally used the per-strike base features rather than the expanded 570 hand-engineered window-level features, because the latter already include manually derived temporal descriptors such as differences, ratios, and summary features that constitute a core part of the proposed feature-engineering approach. This design allowed the CNN-LSTM benchmark to learn temporal dependencies directly from minimally processed sequential inputs.

The CNN-LSTM architecture consisted of a one-dimensional convolutional layer for local temporal feature extraction, followed by an LSTM layer for sequential modeling and a fully connected output layer for binary classification. Hyperparameters were selected based on grouped 5-fold cross-validation performance on the training data, using PR-AUC as the primary selection criterion. The decision threshold was determined from out-of-fold predictions using the same clinically driven strategy as in the main analysis, namely selecting the highest threshold that achieved a target sensitivity of at least 0.80.

The final CNN-LSTM model was then evaluated on the internal validation set and the independent hold-out test set. For cross-validation, 95% confidence intervals for ROC-AUC and PR-AUC were estimated from fold-wise values using the t distribution, consistent with the main analysis. For the independent hold-out test set, 95% confidence intervals for ROC-AUC, PR-AUC, sensitivity, precision, accuracy, specificity, and F1-score were estimated using percentile-based cluster bootstrap resampling at the recording level, consistent with the main analysis.

**Supplementary Results**

In grouped 5-fold cross-validation, the CNN-LSTM benchmark achieved a mean PR-AUC of 0.589 (95% CI, 0.496–0.681) and a mean ROC-AUC of 0.762 (95% CI, 0.729–0.794). The out-of-fold operating threshold was 0.3009, at which sensitivity was 0.801, precision was 0.370, accuracy was 0.612, specificity was 0.550, and F1-score was 0.506.

On the internal validation set, the CNN-LSTM achieved a PR-AUC of 0.620 and a ROC-AUC of 0.793. At the predetermined operating threshold of 0.3009, sensitivity was 0.773, precision was 0.459, accuracy was 0.691, specificity was 0.661, and F1-score was 0.576.

On the independent hold-out test set, the CNN-LSTM achieved a PR-AUC of 0.513 (95% CI, 0.375–0.716) and a ROC-AUC of 0.725 (95% CI, 0.587–0.852). At the predetermined operating threshold of 0.3009, sensitivity was 0.724 (95% CI, 0.545–0.917), precision was 0.412 (95% CI, 0.291–0.543), accuracy was 0.627 (95% CI, 0.471–0.748), specificity was 0.589 (95% CI, 0.381–0.750), and F1-score was 0.525 (95% CI, 0.390–0.641). Overall, the CNN-LSTM benchmark did not outperform the proposed LightGBM model in the current dataset.

| **Supplementary Table 4. Model performance of CNN-LSTM.** | | | | | | | | |
| --- | --- | --- | --- | --- | --- | --- | --- | --- |
| **Dataset** | **ROC-AUC** | **PR-AUC** | **Sensitivity (Recall)** | **Precision (Positive predictive value)** | **Accuracy** | **Specificity** | **F1-score** | **Threshold** |
| Cross-validation (5-fold) | 0.762 [0.729–0.794] | 0.589 [0.496–0.681] | 0.801 | 0.370 | 0.612 | 0.550 | 0.506 | 0.3009 |
| Internal validation | 0.793 | 0.620 | 0.773 | 0.459 | 0.691 | 0.661 | 0.576 | 0.3009 |
| Independent hold-out test | 0.725 [0.587–0.852] | 0.513 [0.375–0.716] | 0.724 [0.545–0.917] | 0.412 [0.291–0.543] | 0.627 [0.471–0.748] | 0.589 [0.381–0.750] | 0.525 [0.390–0.641] | 0.3009 |
| Values in brackets indicate the 95% confidence interval. | | | | | | | | |
